# A conducting polymer with enhanced electronic stability applied in cardiac models

**DOI:** 10.1126/sciadv.1601007

**Published:** 2016-11-30

**Authors:** Damia Mawad, Catherine Mansfield, Antonio Lauto, Filippo Perbellini, Geoffrey W. Nelson, Joanne Tonkin, Sean O. Bello, Damon J. Carrad, Adam P. Micolich, Mohd M. Mahat, Jennifer Furman, David Payne, Alexander R. Lyon, J. Justin Gooding, Sian E. Harding, Cesare M. Terracciano, Molly M. Stevens

**Affiliations:** 1 Department of Bioengineering, and Institute of Biomedical Engineering, Imperial College London, London SW7 2AZ, U.K.; 2 Department of Materials, Imperial College London, London SW7 2AZ, U.K.; 3 School of Materials Science and Engineering, University of New South Wales, Sydney, New South Wales 2052, Australia.; 4 National Heart and Lung Institute, Imperial College London, London SW7 2AZ, U.K.; 5 Biomedical Engineering and Neuroscience Research Group, University of Western Sydney, Penrith, New South Wales 2751, Australia.; 6 School of Physics, University of New South Wales, Sydney, New South Wales 2052, Australia.; 7 Faculty of Applied Sciences Universiti Teknologi Mara, 40450 Shah Alam, Selangor, Malaysia.; 8 National Institute for Health Research Cardiovascular Biomedical Research Unit, Royal Brompton Hospital, London, U.K.; 9 School of Chemistry, Australian Centre for NanoMedicine and Australian Research Council Centre of Excellence in Convergent Bio-Nano Science and Technology, University of New South Wales, Sydney, New South Wales 2052, Australia.

**Keywords:** Conducting polymer, bioelectronic, electronic stability, cardiac, in vivo, ex vivo, optical mapping, bioadhesive

## Abstract

Electrically active constructs can have a beneficial effect on electroresponsive tissues, such as the brain, heart, and nervous system. Conducting polymers (CPs) are being considered as components of these constructs because of their intrinsic electroactive and flexible nature. However, their clinical application has been largely hampered by their short operational time due to a decrease in their electronic properties. We show that, by immobilizing the dopant in the conductive scaffold, we can prevent its electric deterioration. We grew polyaniline (PANI) doped with phytic acid on the surface of a chitosan film. The strong chelation between phytic acid and chitosan led to a conductive patch with retained electroactivity, low surface resistivity (35.85 ± 9.40 kilohms per square), and oxidized form after 2 weeks of incubation in physiological medium. Ex vivo experiments revealed that the conductive nature of the patch has an immediate effect on the electrophysiology of the heart. Preliminary in vivo experiments showed that the conductive patch does not induce proarrhythmogenic activities in the heart. Our findings set the foundation for the design of electronically stable CP-based scaffolds. This provides a robust conductive system that could be used at the interface with electroresponsive tissue to better understand the interaction and effect of these materials on the electrophysiology of these tissues.

## Teaser

Researchers develop sutureless conductive patch with enhanced biostability and effect on heart conduction velocity.

## INTRODUCTION

Among the conductive materials used in bioelectronics, conducting polymers (CPs) have attracted much attention over recent years because of their ability to conduct both electronically and ionically (*1*), to be processed into scaffolds (*2*), and to be rendered biodegradable (*3*, *4*). In tissue engineering, bioelectronic devices are being developed with the anticipation of reestablishing communication between interrupted cells. Low-resistance pathways in excitable tissues, such as the heart, nerves, and muscles, permit rapid cell-to-cell communication via currents generated by the flow of ions between neighboring cells. These low-resistance pathways are critical for the proper function of excitable tissues, for example, electrically synchronizing the cells of the myocardium to produce a functional syncytium (*5*). Disruption in these pathways, for instance, because of myocardium infarction, neuromuscular disorders, and spinal cord injuries, can have a detrimental effect on the tissue. A first hurdle in using organic bioelectronic devices as conductive constructs in tissue engineering is that the CP component would have to maintain its oxidized and electroactive state under physiologically relevant conditions (*6*). Despite the potential of CPs in bioelectronic devices, there is still a challenge of electronic stability required over the lifetime of the implant. This challenge exists because CPs are known to convert from an oxidized (conductive) to a neutral (nonconductive) form upon exposure to air or when in contact with physiological medium (*7*). Another hurdle for the application of some CPs is their poor integration with the host tissue. However, there are some notable successes showing that modulating or adding functionalities in the construct, such as proteins, significantly enhances intimate contact with tissue and thus integration (*8*–*11*). Here, we are interested in addressing the electronic stability of CPs. We describe a novel fabrication approach that immobilizes the “dopant” in the bioelectronic device, generating a polyaniline (PANI) patch that is electronically stable in physiological medium for more than 2 weeks. This finding clears a major hurdle for using PANI, and potentially other CPs, in bioelectronic devices over extended operational times.

The conductivity of CPs stems from a “doping” process through which the polymer is exposed to oxidizing (p-type) or reducing (n-type) agents known as dopants, which are capable of removing or adding electrons to the polymeric backbone (*7*). Specific to PANI, a “proton-doping process” was previously described, where the imine and amine groups of the polymer become protonated upon treatment with a protonic acid; the end result is high conductivities, keeping the number of electrons unchanged (fig. S1) (*12*). This doping process converts CPs from an insulating state to a highly conductive state, and any perturbation in the amount of dopant associated with the polymeric backbone causes changes in the electrical properties of the polymer. Loss of dopant, known as “dedoping,” following incubation under nonacidic or physiological conditions is a severe limiting factor for the long-term use of conjugated polymers in biological and medical applications (*7*). Significant loss of dopant accompanied by a reduction in conductance occurs from polypyrrole films within 2 days of incubation under physiological conditions (*13*). Similarly, polythiophene-based biomaterials are converted to the reduced form following incubation in cell culture medium (*14*) or through exposure to air (*15*). A further challenge for PANI is the deprotonation of amine groups caused by the neutral pH of bodily fluid (*16*, *17*). In a study by Gill *et al.* (*18*), a reduction in conductance was directly related to the level of deprotonation of the PANI amine groups that occurs in response to neutral or higher pH solutions.

Here, we describe a new fabrication process based on polymerizing aniline in the presence of the dopant phytic acid on a prefabricated chitosan film that meets the challenge of deprotonation. Taking advantage of the multivalent anionic nature of phytic acid and the positively charged amine groups on both the chitosan and the PANI backbone, the PANI/chitosan patch is designed to retain the dopant and thus remains in the conductive state, as we herein demonstrate by applying different characterization techniques. Whereas chitosan is an insulator, its use as a substrate is advantageous to produce a flexible and mechanically robust freestanding conductive film without compromising the electronic activity. The ease of the fabrication technique used is of significance for the application of this film as a polymeric patch in tissue engineering. This is in contrast to hard electronics, which have a significant mismatch in mechanical properties with tissue (*1*). Together with the in vitro electronic biostability, this freestanding conductive patch serves as a platform to interface with electroresponsive tissues such as the heart. We investigated the effect of the patch on cardiac electrophysiology using both cardiac slices and Langendorff-perfused hearts. A further benefit of using the chitosan film is that it could be applied to tissue without the need for sutures. Upon the addition of Rose Bengal (RB), a photoactivated dye, the chitosan film can operate as a bioadhesive (*19*, *20*). The RB-chitosan film adheres to tissue following irradiation with a green laser (λ = 532 nm), avoiding the more commonly used surgical suturing. Here, we used this technology to produce a sutureless conductive patch that was implanted successfully in vivo for 2 weeks.

## RESULTS AND DISCUSSION

### Fabrication of the PANI patch

**Fig. 1:**
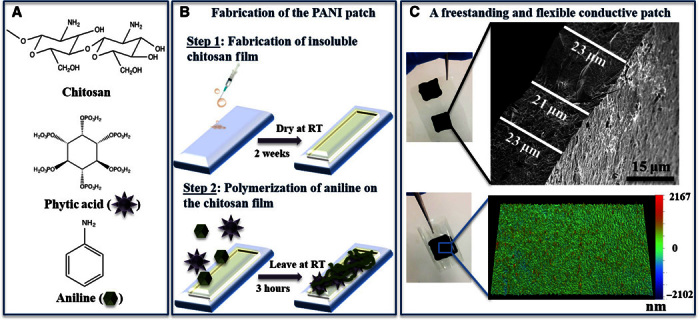
Schematic representation of patch fabrication. Chemical components (**A**) and fabrication steps (**B**) used to produce the PANI patch. RT, room temperature. (**C**) Top: SEM micrograph of a cross section of the patch. Bottom: Surface topography of the patch recorded using optical profilometer.

The PANI patch was fabricated in a two-step procedure (Fig. 1, A and B). First, chitosan solution was drop-casted on a glass slide to form a well-defined film that was left to dry for 2 weeks. This drying process is simple and scalable: No chemical modification is required for the fabrication of totally insoluble chitosan films (*21*, *22*). The second step was drop-casting the mixture of aniline, phytic acid, and ammonium persulfate (APS) on the surface (*23*). The change in color from light brown to green indicated the onset of aniline polymerization, which eventually transformed to a thick layer of metallic green polymer on the surface. After 3 hours, the reaction was quenched with water, and a superficial layer of PANI was washed away, with only the PANI crosslinked to the chitosan surface remaining. Phytic acid has six phosphonic acid groups (PO_3_H_2_), and its proton dissociation falls in three regions (*24*), leading to a negatively charged compound for pH >2. With its high number of anions, phytic acid can protonate the amine groups on the chitosan backbone (*25*) and also binds to the positively charged PANI (*23*). The chitosan layer functions as a base membrane binding strongly to phytic acid, which, in turn, bonds to the PANI chains, resulting in a blended system with strong interactions between its three different components. Ionically crosslinking the dopant phytic acid with both chitosan and PANI has the advantage of creating a system with far greater dopant stability.

### In vitro characterization of the patch’s physical properties

The fabricated patch was peeled off the glass slide to produce a freestanding patch with a thickness of 19.06 ± 3.36 μm, as determined by scanning electron microscopy (SEM) (Fig. 1C), in comparison to 12.10 ± 2.07 μm for unmodified chitosan films. The thickness of the patch can be easily tailored to suit different requirements by simply changing the volume of the drop-casted chitosan solution. The microscale topography of these patches imaged by optical profilometry (Fig. 1C) is uniform over a large surface area (117 μm × 156 μm; fig. S2), indicating the suitability of the drop-casting technique to dispense the phytic acid/aniline solution followed by its polymerization. The mechanical properties of the patch (Young’s modulus, 6.73 ± 1.14 MPa; tensile strength, 5.26 ± 2.25 MPa; elongation at break, 79 ± 22%; Table 1) are comparable to values reported for other PANI composites, such as poly(glycerol sebacate) containing 20 and 30% PANI (*26*). The contact angle of the patch of 62.1 ± 1.4° suggests a moderate wettability (48° to 62°) (*27*). In comparison to other PANI composites (*28*), the surface of the patch exhibits a higher hydrophilicity because of the phosphonic groups in phytic acid.

**Table 1:** Physical properties of the PANI patch.

**Physical property**	**Measured value**
Mechanical properties	
Young’s modulus	6.73 ± 1.14 MPa
Ultimate tensile strength	5.26 ± 2.25 MPa
Elongation at break	79 ± 22%
Contact angle Thickness	62.1 ± 1.4° 19.06 ± 3.36 μm

### In vitro characterization of the patch’s electronic properties

#### Cyclic voltammetry.

**Fig. 2:**
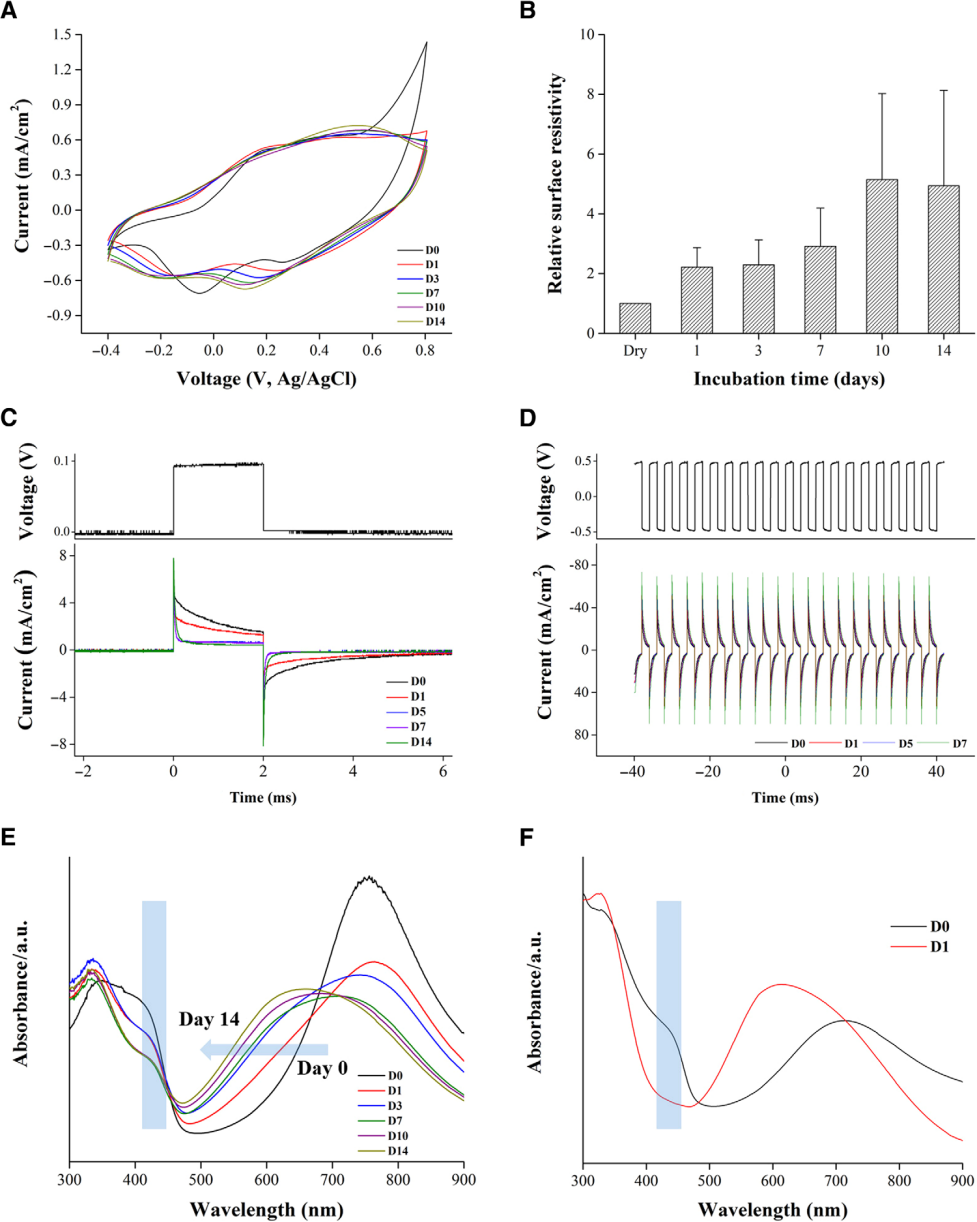
Electronic stability of PANI patches demonstrated in vitro over 2 weeks of incubation (A to E) and UV spectrum of PANI-PCL film (F). (**A**) Cyclic voltammograms (25 mV·s^−1^) in 0.1 M PBS reveal the stable electrochemistry. D, day. (**B**) Surface resistivity exhibits only a fivefold increase in value. Continuous electric stimulation (**C** and **D**) reveals that the patch retains a constant current after 2 days of incubation in buffer. Ultraviolet-visible (UV-Vis) spectra of (**E**) the PANI patch and (**F**) PCL-PANI as a function of incubation time in PBS (pH 7.4): The peak at 420 nm persists in our PANI patch, whereas it disappears after 1 day of incubation in the PCL-PANI film, indicating conversion to the nonconductive form. a.u., arbitrary units.

Cyclic voltammetry was recorded in phosphate-buffered saline (PBS; 0.1 M, pH 7.4) at a scan rate of 25 mV·s^−1^ versus Ag/AgCl (3 M KCl) (Fig. 2A). Immediately after washing, an obtained cyclic voltammogram exhibited characteristic features of PANI, indicated by two redox couples: a primary couple at 0.38 V (anodic) and 0. 28 V (reduction) and a secondary couple at −0.20 and −0.05 V. Despite the fact that the cyclic voltammetry was recorded in buffer (pH 7.4), its characteristics are typical of PANI in pH <6.0 (*29*) because of the internal acidic environment induced by the phytic acid molecule. After 1 day of incubation in buffer, we noticed a slight increase in peak separation between the anodic and cathodic peaks of each redox couple, indicative that charge transfer was becoming more difficult. At days 7 and 14, only the primary redox couple appeared as expected at more alkaline pH (fig. S3) (*29*), accompanied by a cathodic shift. Nevertheless, the current remained stable over the whole incubation period in neutral pH, unlike PANI films doped with conventional small acids that exhibit negligible electrochemical redox activity at pH >4 due to loss of dopant (*30*, *31*). The electrochemical behavior observed for the patch in PBS indicates that crosslinking a small multivalent dopant, such as phytic acid, during the synthesis of PANI is a viable approach to producing conductive materials that are electroactive under physiologically relevant conditions over extended periods of time. Although the patch comprised a significant amount of the insulator chitosan (chitosan film uncoated with PANI; surface resistivity, >10^2^ megohms per square; fig. S4), it appears that effective charge transport can occur through the patch even in PBS as an electrolyte.

#### Surface resistivity.

To test how the surface resistivity of the patch varied with incubation in pH 7.4 as a function of time, patches were assembled into custom-made chambers and their current-voltage (*I-V*) traces (two-probe) were recorded when they were still in the dry state (surface resistivity, 7.93 ± 2.64 kilohms per square); this was followed by adding PBS and incubating at 37°C. At different time points, the PBS was removed and the patch was gently blotted dry before the *I*-*V* curves were recorded. After 1 day of incubation, the surface resistivity doubled in value before stabilizing (Fig. 2B). A further increase (five times the original value) was recorded at days 10 and 14 (35.85 ± 9.40 kilohms per square). The increase in patch resistance was attributed to partial loss of dopant, which led to a lower protonation level of PANI [as will be shown from x-ray photoelectron spectroscopy (XPS) data]. Nevertheless, the system appeared to have markedly enhanced stability compared to other PANI-based biomaterials, which exhibited a several orders of magnitude increase in surface resistivity after they were exposed to buffer (~6 megohms per square after 100 hours of incubation in cell culture medium) (*32*). Extending the lifetime of PANI-based bioelectronics in the conductive form has the potential to have a significant impact in bioapplications that require prolonged electrical stimulation regimes, for instance, in cardiac regeneration (*33*). Here, the PANI patch is capable of withstanding trains of electric pulses mimicking those of the adult heart (*33*, *34*) for 2 weeks in cell culture medium, with the patch as an anode and the Pt plate as the cathode. As shown in Fig. 2C, from day 5 onward, the current generated remains constant for the whole duration of continuous stimulation. By conditioning the patch in cell culture medium for 2 days, similar electronic stability was verified by subjecting the patch to pulses of ±0.5 V (Fig. 2D). The low surface resistivity exhibited by the patch developed here allows this material to act as a conductor under physiological conditions for at least 2 weeks.

#### UV-Vis spectroscopy.

For an understanding of the effect of incubation on the electronic structure of the patch, samples were incubated in PBS and their UV-Vis spectra were recorded at predetermined time points (Fig. 2E). Changes in the protonation and oxidation states of PANI could be monitored by observing three characteristic absorbance peaks: 320 nm (π-π* transition of benzenoid group), 420 nm (polaronic shoulder), and 500 to 900 nm (polaron region). Of significance is the peak at 420 nm assigned to the emeraldine salt (fig. S1), the electrically conductive form of PANI (*29*). The spectrum of the patch at fabrication showed a broad flat peak in the region between 345 and 420 nm, suggestive of a doped polymer (*35*). As expected, another broad peak appeared in the region of 750 to 850 nm, which is typical of the emeraldine salt form. After 1 day of incubation, a distinct peak appeared at 330 nm and a shoulder at 420 nm. This suggested that the level of doping decreased as a result of incubation in buffer, which agrees with the surface resistivity and electric stimulation recordings at day 1. To assess the effect of longer incubation times on the protonated state of the patch, we monitored the peak at 420 nm (fig. S5A) and the shift of the maxima in the polaron region between 500 and 900 nm (fig. S5B). As the incubation time increased, a decrease in the 420-nm peak was observed. This was accompanied by a blue shift in the polaron region, which suggests that the patch was converted from the emeraldine salt form to the emeraldine base (nonconductive) form. After 10 days in buffer, the polaron region peak became centered at 650 nm (fig. S5B), suggesting that the emeraldine base form becomes the dominant state of the patch. The changes observed in the spectral characteristic peaks agree with changes in both the cyclic voltammetry and the surface resistivity (Fig. 2, A and B). The UV data suggest that the bulk of the patch maintained a protonated species after 2 weeks in buffer, as demonstrated by the presence of the 420-nm peak in all measured spectra.

To investigate whether the resistance of the patch for complete deprotonation in neutral buffer was due to our fabrication design of crosslinking the dopant to a base material, we fabricated a polycaprolactone PANI blend (PCL-PANI) doped with a conventional small acid, camphor sulfonic acid (CSA) (*36*), and characterized its UV spectra (Fig. 2F) in response to buffer. We chose this system because we could drop-cast the mixture to produce freestanding films of similar thickness to ours. At fabrication, the UV spectra showed the typical characteristics of a doped PANI: The polaron peak is centered at 740 nm along with a distinctive peak at 420 nm corresponding to the emeraldine salt form of PANI. After 1 day of incubation, the UV spectra showed that the PCL-PANI film is completely converted to the emeraldine base form of PANI: The 420-nm peak disappeared, and there was a shift of the polaron peak to 625 nm. This is due to the leaching out of the dopant from the film as it comes into contact with the buffer, which causes the PCL-PANI to convert from a conductive to a nonconductive form in a very short time (<24 hours). This result confirms that our approach of crosslinking the dopant with the polymers leads to a PANI patch that exhibits a conductive nature over extended periods (14 days versus 1 day) under physiological conditions.

#### X-ray photoelectron spectroscopy.

**Fig. 3:**
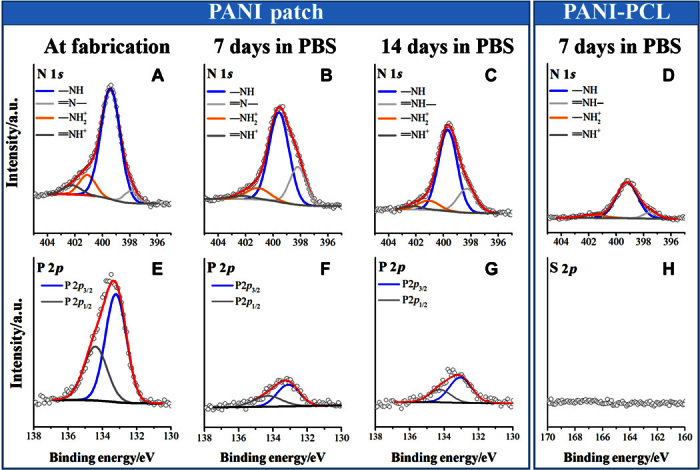
Detection of protonated species as a function of incubation time. High-resolution narrow scans of N 1*s* (**A** to **D**), P 2*p* (**E** and **F**), and S 2*p* (**G**) core-line spectra of the PANI patch at fabrication (A and E) and incubated for 7 days (B and F) or 14 days (C and G) in buffer. N 1*s* (D) and S 2*p* (**H**) core-line spectra of PCL-PANI incubated for 7 days in buffer. Peak heights are normalized to the peak maxima of the N 1*s* (A to D) or P 2*p* (E to G) of the patch at fabrication (first column). Within each row, panels are on the same arbitrary *y* axis.

XPS measurements were conducted to detect any changes in the oxidation state and doping level of the patch that might have occurred as a result of incubation in buffer. The C 1*s*, O 1*s*, N 1*s*, and P 2*p* core-level spectra were analyzed at fabrication (day 0) and after 7 and 14 days of incubation (Fig. 3 and fig. S6). The residing state of nitrogen in the PANI backbone is of particular interest because its chemical environment reveals the oxidation and protonation states of PANI (fig. S1) and can be related to the doping level induced by the addition of acids (*17*, *35*). To this effect, the relative amounts of protonated imine and amine were estimated from curve-fitting the N 1*s* spectra (Fig. 3, A to D). The chemical interactions in the patch led to the assignment of four N chemical environments: imine at ~398.2 eV, amine at ~399.7 eV, oxidized amine (–NH^+^) at ~401.0 eV, and protonated imine (=NH^+^) at ~402.0 eV. Table 2 lists the percentage of each nitrogen species calculated by comparing the peak area of individual nitrogen species to the total N 1*s* area (*37*, *38*).

All samples contained two species of the positively charged nitrogen, –NH^+^ and =NH^+^, which can be assigned as polaron and bipolaron states, respectively, as previously reported (*39*). These positively charged species correlate to the doping level of PANI, and their relative amounts varied as a function of incubation time. At fabrication, the patch exhibited a doping level of ~18%, in agreement with doping levels reported for electropolymerized PANI films (*40*) and PANI colloids prepared by chemical oxidation in acidic medium (*41*). However, some PANI systems have been reported to have higher doping levels (*35*), for instance, as is the case for the PCL-PANI blends fabricated in this study, with a doping level of ~40% (Table 2 and fig. S7). The lower doping level in our patch is likely due to two factors. First, the patch has a lower dopant-to-monomer ratio than PCL-PANI, and thus, one expects a lower amount of dopant to be incorporated into the former. Second, strong intermolecular interactions can occur between the multivalent anion phytic acid and the amine groups of chitosan (*25*) and therefore reduce the availability of phytic acid as a dopant to PANI. Despite the lower protonation level, the patch exhibits a high conductivity value of 0.162 ± 0.043 S/cm.

The ratio of the positively charged N decreased as incubation time increased, which is consistent with the well-known deprotonation of PANI in buffer (pH 7.4). However, after 2 weeks of incubation, the patch retained ~12% of the protonated species. This result agreed with the UV spectrum (day 14; Fig. 2E), which exhibits peak characteristics corresponding to the emeraldine salt form of PANI. In comparison, the PCL-PANI blend had decreased to only ~6% protonation within a single week of incubation. We attribute the enhanced stability of the protonated species in our patch to the multivalent anionic nature of phytic acid and its ability to bind strongly to chitosan and PANI. In contrast, CSA within the PCL-PANI blend has one negative charge and can easily diffuse out of the film, as previously reported for similar systems doped with conventional small-molecule acids (*13*).

Complementary information about the change in the level of doping state in response to incubation was obtained by tracking the element phosphorous (P). One P 2*p* chemical environment consisting of the two spin-orbit split states (P 2*p*_3/2_ and P 2*p*_1/2_) was curve-fitted with a spin-orbit splitting of 1.1 eV (Fig. 3, E to G). In agreement with the trend observed for the protonated species, its value decreased after incubation. However, the detection of P after 14 days of incubation suggests that some of the dopant was retained on the surface of the patch, explaining the protonated species detected by XPS. By contrast, monitoring the sulfur component in the PCL-PANI films revealed that the dopant CSA did not exist on the surface after 7 days in buffer (Fig. 3H), in agreement with the loss of the protonated species (UV and XPS data). This result agrees with that from Fonner *et al*. (*13*), who showed the disappearance of small dopants, such as chloride and *p*-toluene sulfonate ions, from electropolymerized polypyrrole films after 2 days of incubation in PBS. Similar dopant loss has been shown by Mahat *et al*. (*17*).

**Table 2:** Elemental ratios by XPS surface analysis.

	**% N 1*s* signal**						
	**Incubation** **time** **(days)**	**=N−**	**−NH**	**−NH_2_^+^**	**=NH^+^**	**P 2*p*_3/2_/** **C 1*s***	**S 2*p*_3/2_/** **C 1*s***
**PANI patch**	0	6	77	12	6	0.014	
	7	23	65	10	3	0.003	
	14	21	68	9	3	0.004	
**PCL-PANI**	0	10	51	32	7.7		0.01
	7	11	90	6	Negative		Negative

### The patch tested in an ex vivo cardiac model

The electronic biostability of the PANI patch under physiological conditions points encouragingly to application with electroresponsive tissues. An obvious application is the heart because many cardiac diseases result in disruption of the electrical signal of the heart wall. Cardiac patches have been shown to have a beneficial effect on cardiac function and reconstruction of the damaged tissue (*42*). More recently, there is emphasis on developing cardiac scaffolds that have the potential to electrically couple with the heart. Conductive materials used in the fabrication of these scaffolds include conjugated polymers (*26*, *43*, *44*), carbon-based materials such as graphene/graphene oxide (*45*, *46*) and carbon nanotubes (*47*–*49*), and metallic nanoparticles or nanowires such as gold (*50*). Introducing conductivity in these scaffolds resulted in several benefits: enhanced electrical coupling (*47*, *48*, *50*), higher rates of spontaneous beatings of cardiomyocytes cultured on these scaffolds (*46*), and improved function of infarcted hearts (*45*, *49*). Having established that our patch sustains its electronic properties in vitro, we set out to investigate in the first instance whether its attachment to cardiac tissue will have any effect on the mechanical or electrophysiological function. We conducted ex vivo examinations on both ultrathin cardiac slices and whole hearts.

#### Cardiac slices.

**Fig. 4:**
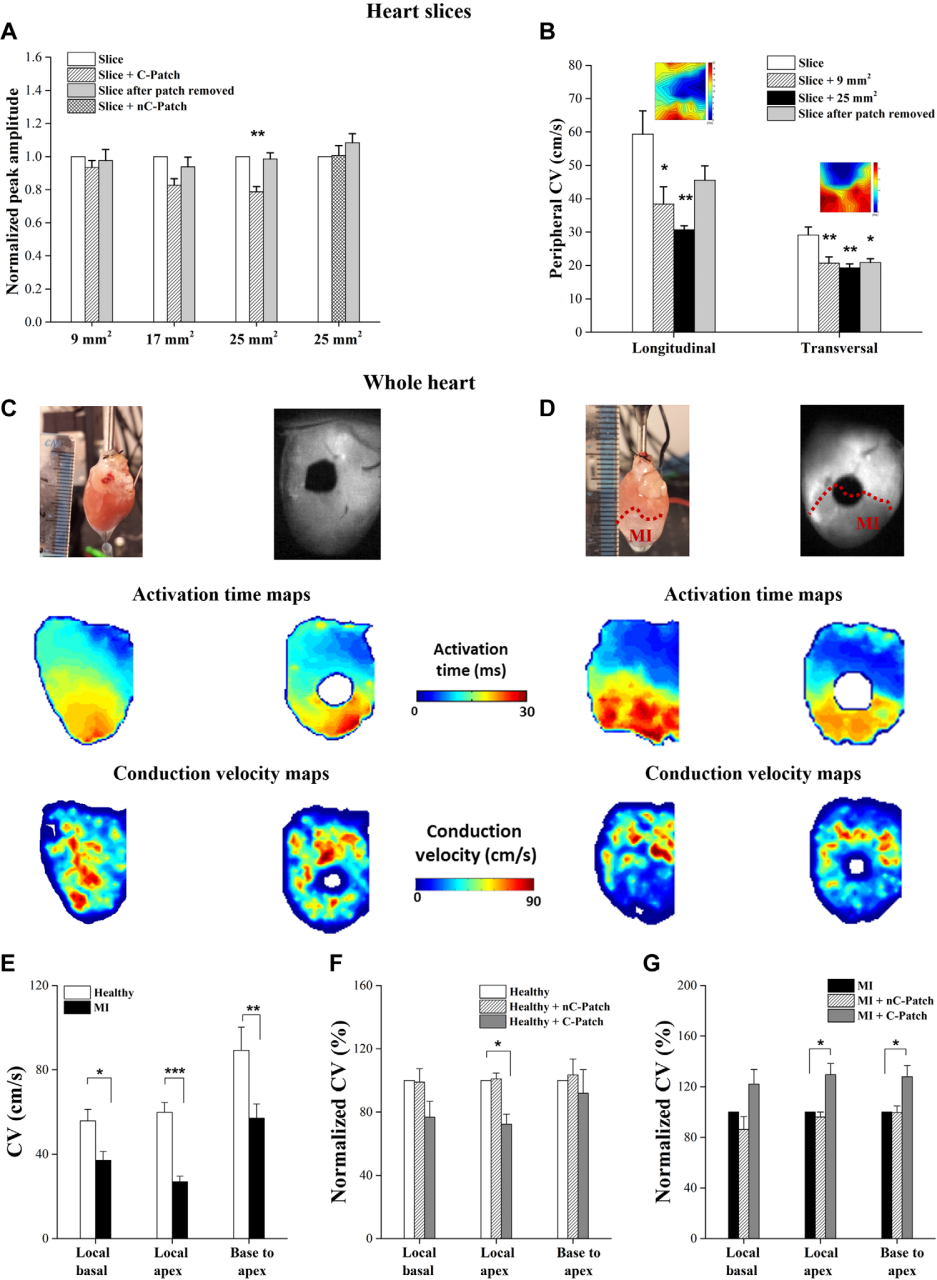
Ex vivo and in vivo cardiac evaluation of the PANI patch. (**A**) Contractility of the cardiac slices in response to field stimulation. (**B**) Conduction properties of cardiac slices measured by MEA system. (**C** to **G**) Ex vivo whole-heart epicardial voltage mapping: Representative activation time and CV maps before (C) and after (D) conductive patch application on healthy control hearts (C) and on hearts 2 weeks after MI (D) [permanent ligation of left anterior descending coronary artery (LAD)]. (E) CV in healthy and MI hearts (*n* = 10, *P < 0.05, **P < 0.001, ***P < 0.0001). (F) Normalized CV in healthy hearts before and after patch application (*n* = 5, *P < 0.05). (G) Normalized CV in MI hearts before and after patch application (*n* = 5, *P < 0.05). “Local basal”: average CV in basal half of the heart (noninfarcted area) calculated on a pixel-by-pixel basis from the activation time maps; “Local apex”: average CV in apical half of the heart (infarcted area); “Base to apex”: CV calculated across whole heart from pacing electrode to apex.

Conductive patches (C-Patches) of different sizes were positioned on cardiac slices, and the contractility of the tissue was measured in response to field stimulation (Fig. 4A). Slices that had C-Patches of 9 and 17 mm^2^ in size (covering 22.5 and 42.5% of the slice surface area, respectively) exhibited contractile forces comparable to those that had no patch, which indicates that the slices remain electrically active upon stimulation. However, as the C-Patch size was increased to 25 mm^2^ (covering ~63% of the slice surface area), a significant drop in the amplitude was observed (~21%, *P* < 0.05, *n* = 13). Subsequently, upon removal of the patch, the slice showed complete recovery of contractility. To confirm that this drop in contractility is not a mechanical effect induced by the patch but rather due to its electronic properties, the contractility of slices was measured in the presence of a nonconductive patch (nC-Patch) with a similar Young’s modulus (6.99 ± 2.58 MPa). The contractile forces were found to be comparable to the contractile force of the slice before the nC-Patch was added or after it was removed.

The effect of the conductive patch on the electrophysiology of the heart slices was assessed using the multielectrode array (MEA) system (*51*). The conduction properties were determined first for the heart slice at baseline, followed by positioning the C-Patch (9 or 25 mm^2^) on the top side of the slice, and finally after the C-Patch had been removed. Figure 4B presents a summary of the average longitudinal and transverse conduction velocities (CVL and CVT, respectively) obtained. The slices exhibit a higher CVL than CVT, as expected, reflecting the electrical anisotropy of the cardiac tissue. A significant drop in CVs was observed for both CVL and CVT when the C-Patch was positioned on the slice, irrespective of its size. The patch was removed, and an immediate measure of the slice CV was recorded. In the case of CVL, the CV of the slice was regained after C-Patch removal; however, this recovery was not observed in the CVT.

#### Whole hearts.

Optical mapping experiments were conducted to probe the effect of the C-Patch on the electrophysiology of whole hearts (Fig. 4, C to G) (*52*, *53*), studied ex vivo and taken from both healthy animals and those in which a myocardial infarct (MI) had been induced by coronary ligation, and allowed to develop for 2 weeks. Epicardial optical mapping generated activation time maps, and subsequently CV maps, of healthy (Fig. 4C) and MI hearts (Fig. 4D) before and after the application of the patch. In agreement with the results obtained from the slices, apical CV dropped significantly when the C-Patch was sutured to the healthy hearts (66.3 ± 4.3 cm/s to 47.8 ± 5.1 cm/s, *P* < 0.02, *n* = 5), whereas no significant effect on the CV was observed following the attachment of nC-Patch. CV was significantly lower in the infarcted hearts compared to the healthy hearts as expected, and this was most pronounced in the apex (26.9 ± 2.7 cm/s versus 59.8 ± 4.7 cm/s, respectively, *P* < 0.001, *n* = 10). The C-Patch increased CV in the apical infarcted area (24.3 ± 4.9 cm/s to 30.1 ± 4.2 cm/s, *P* < 0.05, *n* = 5). The change in CV was significantly different between healthy and MI hearts after conductive patch application [*P* < 0.01, one-way analysis of variance (ANOVA) with Dunn’s post hoc test]. Action potential duration (APD) was only minimally affected by the presence of both types of patches and is unlikely to have a role in the observed effects (fig. S8).

Therefore, attaching the C-Patch on either heart slices or whole hearts has an effect on cardiac electrophysiology, whereas the nC-Patch, an insulating material, did not interfere with the cardiac electric activity. Although the exact mechanism is not elucidated at present, this points to the exciting fact that it is the electroactive nature of the patch, and not its mechanical effect, which is the factor in the observed changes.

### In vivo application of the patch

One could envision that the clinical application of the C-Patch may require that the material be positioned on both healthy and damaged tissue (as in Fig. 4D), in particular, if the patch is ultimately to be used to manipulate the electrical properties of damaged areas of the heart. Although patches are considered to have some advantages over injectable cells/hydrogel systems in that they retain more cells of the delivered dose and provide structural support, their implantation on the heart has always been looked at as challenging and complex (*42*) and often requires invasive suturing techniques. Here, we introduced a new sutureless method for adhesion of patches onto heart tissue (movie S1), namely, using photoadhesion with a green laser (fig. S9 and movies S1 and S2) (*54*, *55*). Consequently, we conducted a preliminary in vivo study to investigate whether the sutureless C-Patch has any effect on cardiac function and whether it imposes a proarrhythmic risk. Furthermore, we wanted to test whether the photoadhesion was effective in securing the patch onto a beating heart despite abrasive effects from contents of the intrathoracic cavity.

#### Effect on cardiac function.

**Fig. 5:**
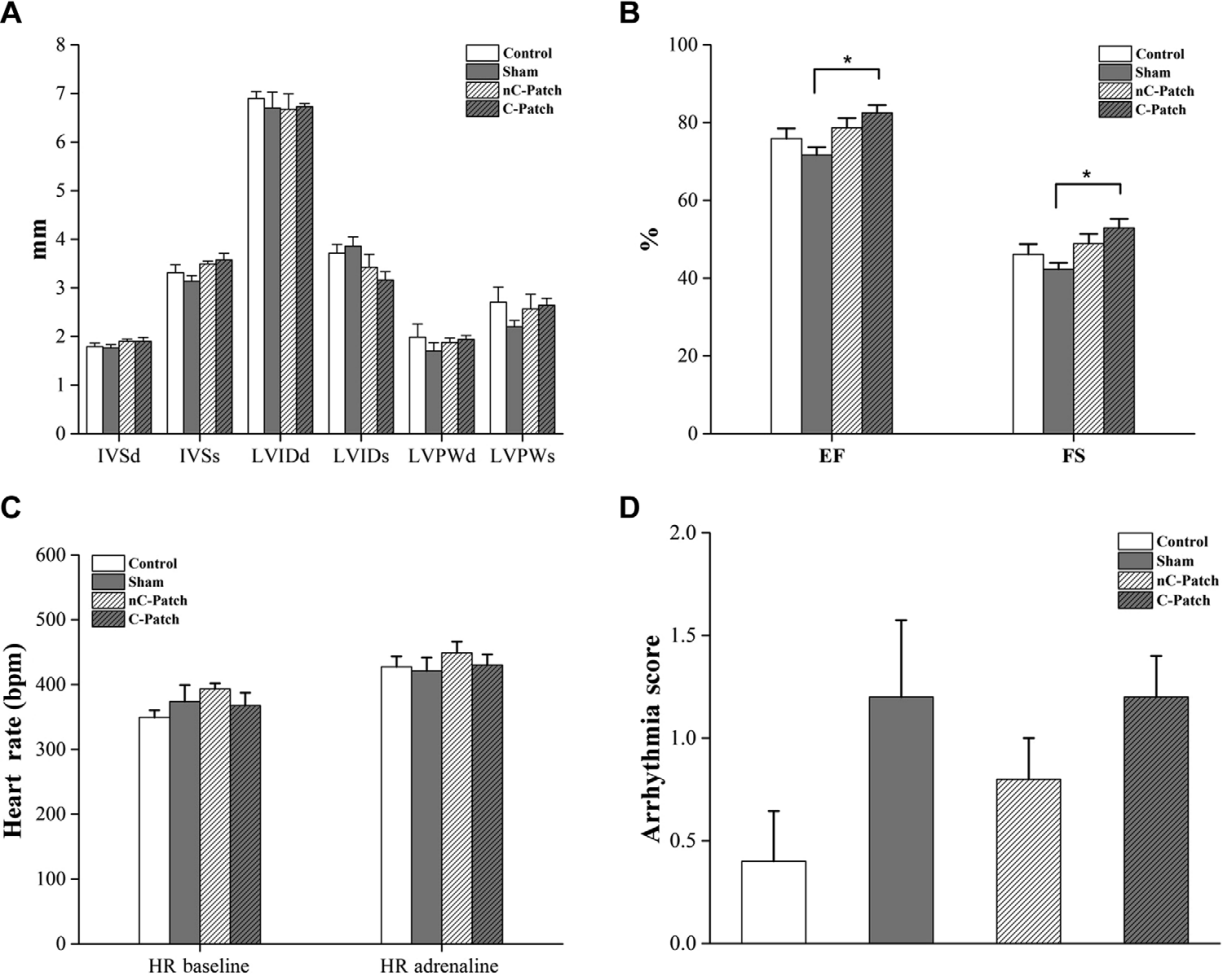
Echocardiographic assessment of cardiac function and arrhythmia provocation 2 weeks after patch implantation. (**A**) Left ventricular dimensions. (**B**) Left ventricular function (*n* = 5; *P < 0.05). IVS, interventricular septum; LVID, left ventricular internal diameter; LVPW, left ventricular posterior wall; d, diastole; s, systole. (**C** and **D**) Rats were given two intraperitoneal injections of adrenaline (0.5 mg/kg) to induce arrhythmias, with the electrocardiogram (ECG) recorded for 30 min after each injection. (C) Average heart rate (HR) over 10 min recorded before adrenaline injections (baseline) and 20 min after the second adrenaline injection. (D) Each animal is assigned an arrhythmia inducibility score: 0 = no arrhythmias; 1 = only minor events such as premature ventricular contractions (PVCs), couplets, and triplets; 2 = more severe events such as nonsustained VT, sustained VT, and VF.

Echocardiography results showed that adhesion of the patches (both nonconductive and conductive) had no significant effect on global heart function (Fig. 5, A and B). A small but a significant increase in the ejection fraction (EF) and fractional shortening (FS) of hearts with C-Patches was observed in comparison to the sham group (Fig. 5B). This could not be ascribed to differences in chest wall adhesions of the patch because the degree of adhesion was consistent between the groups. The relaxation properties of the heart (left ventricular internal diameter in diastole, Fig. 5A) were unaffected by either patch. This suggests that the patches, despite having a higher Young’s modulus than typical adult myocardium, do not inhibit the contractile properties of the heart. A series of experiments involving administration of adrenaline (0.5 mg/kg, intraperitoneally) was conducted to examine whether the C-Patch predisposes to arrhythmia. Heart rate increased in all groups after adrenaline injection (Fig. 5C), as would be expected. However, this increase was comparable between all four groups. Injection of adrenaline induced arrhythmic events [ventricular ectopics, nonsustained ventricular tachycardia (VT), VT, ventricular fibrillation (VF)] in all experimental groups. No significant difference in arrhythmia score was observed between the four groups (Fig. 5D). These results suggest that patches applied by photoadhesion that are conductive in nature do not influence the proarrhythmic state of the heart under stress and may be safe in cardiac application.

#### Sutureless application of the patch and immunohistology.

After 2 weeks in vivo, the patches remained adhered to the cardiac tissue (fig. S9C), which indicated that photoadhesion is an effective technology (movie S2) to apply patches to the heart in a potentially less invasive manner. It is reported in the literature that the adhesion is due to a photocrosslinking mechanism between biomolecules initiated by the activation of RB into an excited state (*56*, *57*). Further investigations involving spectroscopy techniques are needed to elucidate the exact molecular mechanism underlying the bonding between chitosan films and tissue via green lasers. To examine the interaction of the patch with the myocardial surface, histology was performed 2 weeks after implantation (figs. S10 and S11). Both the C- and nC-Patches were surrounded by a cellular infiltrate, which continued around the heart, decreasing in thickness in relation to the distance from the patches (fig. S10, A to D). The thickness of encapsulation was comparable between both patches, as shown in fig. S11E. Similar thickness has been reported for a porous poly(glycerol sebacate) scaffold tested as a cardiac patch after 4 weeks of implantation (*58*).

Immunostaining for vimentin and CD45 (fig. S10, H and I) revealed that the cell population was composed of fibroblasts (vimentin^+^) and inflammatory cells (CD45^+^). In the fibrotic tissue surrounding the patch, there was some neovascularization indicated by von Willebrand factor staining (fig. S10, E to G). Although the patches were not in direct contact with the myocardium, the surface appeared undamaged because the border myocardium/scar was well defined and the myocytes in proximity to the fibrotic scar were well organized (fig. S10, L to N, and fig. S11). This also indicates that the laser irradiation used for photoadhesion at the time of implantation did not cause damage to the cardiac tissue. Photoadhesion using RB either as a solution or embedded in a biomaterial, such as chitosan, has been extensively applied in vitro and in vivo without any adverse reactions or cytotoxicity effect on the surroundings (*19*, *54*, *55*). In addition, photoadhesion using RB has been tested in a clinical trial that included 27 patients (*59*), with minimal inflammation reported during the early healing process, attributing this to a lack of phototoxicity (*60*). Sham controls showed only a limited cellular expansion of the epicardium in comparison to control rats (fig. S10, A, B, E, F, L, and M). Smooth muscle actin expression is a well-known marker for fibroblast activation and differentiation into myofibroblasts; cells expressing this marker were noticed in the encapsulation of the heart and in near proximity to both the C- and nC-Patches (fig. S10, J and K). It is clear that, for the C-Patch to be potentially applied as a cardiac patch, design strategies targeting the material-cell interface should be developed to modulate the immune response, such as the loading of anti-inflammatory mediators or alteration of the surface topography and roughness (*61*). Integration of conductive scaffolds has been successfully shown in the literature (*8*–*11*), and this should inspire future work to enhance the adhesion of the patch to biological tissues.

## CONCLUSIONS

In summary, we have shown that, by immobilizing the dopant in the conductive construct, enhanced electronic stability under physiologically relevant conditions can be achieved. After 2 weeks of incubation in buffer, the patch exhibited stable electrochemistry and relatively low surface resistivity, and its UV characteristics were those of the emeraldine salt form of PANI. These results were further confirmed by XPS, which revealed the presence of protonated species on the surface over the incubation period. New insights into the effect of conjugated polymers at the interface of cardiac tissue have been obtained by applying the conductive patch in ex vivo cardiac models. An interesting observation was the effect of the conductive patch on the CV of the heart, lowering its value in healthy models while increasing it in infarcted hearts. Future work should focus on unraveling the mechanism behind this effect. In vivo studies indicated the safe sutureless application of the C-Patch on cardiac walls. The stability of this patch in the conductive state under physiological conditions and its demonstrated effect on cardiac electrophysiology sets the foundation for the design of electronically stable CP-based scaffolds that could effectively function as electronic interfaces. Significantly, this study presents a robust bioelectronic device that, because of its electronic stability, could potentially be applied at the biotic-abiotic interface to elucidate the role of conductive materials in electroresponsive tissues in both ex vivo and in vivo models.

## MATERIALS AND METHODS

### Materials

Chitosan (medium molecular weight, 85% deacetylation), RB, aniline, phytic acid [50 weight % (wt %) in water], APS, PCL, CSA, hexafluoroisopropanol, acetic acid, and phosphate buffer tablets were all purchased from Sigma-Aldrich and used without further purification. Chemicals for the ex vivo experiments were purchased from either VWR International or Sigma-Aldrich.

### Experimental methods

#### Fabrication of PANI patch.

The PANI patches (C-Patch) were fabricated by first preparing a chitosan film followed by the polymerization of aniline on the film surface in the presence of phytic acid as a dopant (*23*). In a typical procedure to produce the nC-Patch, chitosan films were prepared, as described by Lauto *et al*. (*19*), without the addition of RB. Chitosan (medium molecular weight, 85% deacetylation) was dissolved at 1.8 wt % in 2% (v/v) acetic acid solution. The solution was centrifuged at 4000 rpm for 5 min to get rid of any insoluble flakes. Chitosan solution (1.6 ml) was evenly spread on a microscope slide (7 cm × 2 cm) and left to dry for 2 weeks at atmospheric pressure. To prepare a C-Patch (1 cm × 1 cm), two solutions were prepared. Solution 1 contained phytic acid (2.30 ml) and aniline (1.15 ml) in deionized water (DI H_2_O) (5 ml). Solution 2 contained the oxidant APS (1.42 g) in DI H_2_O (5 ml). Varying the molar ratio of aniline to the dopant or aniline to the oxidant resulted in no significant change in surface resistivity (see table S1). The two solutions were left in the refrigerator for 30 min before use. Solutions 1 (77 μl) and 2 (23 μl) were mixed in an Eppendorf tube, vortexed, and then dispensed on the surface of the chitosan film. The solution was allowed to polymerize on the surface for 3 hours. To quench the polymerization reaction, patches were rinsed with DI H_2_O thoroughly until no PANI flaked off the surface. Patches were then incubated in DI H_2_O overnight to wash out any excess phytic acid, uncrosslinked polymeric chains, and any side products. Patches were then peeled off the glass slide, blow-dried with N_2_, and stored between two glass slides to maintain their flat shape. Care was taken to ensure that the top side of the patch was properly identified. All characterizations were done on the top side of the patch. Blends based on PCL and PANI doped with CSA were fabricated according to the procedure described in the literature (*36*). To obtain films of comparable thickness to the PANI film, 1.6 ml of the PCL-PANI solution was evenly spread on a glass slide (7 cm × 2 cm) and left to dry for 2 days at room temperature. For the in vivo experiments, the C-Patches contained RB needed for photoadhesion. RB (0.1 wt %) was dissolved in the chitosan solution, and the same procedure was repeated as described above. The geometry of these patches is shown in fig. S9C.

#### Characterization of physical properties.

The thickness was determined by imaging the edges of the patches (*n* = 5) using SEM operated at 15 kV. The mechanical properties were determined using a calibrated single-column tensiometer that was interfaced with a PC (Instron). Patches (3 mm × 5 mm, *n* = 10) were kept moist before the tensile test to mimic in vivo conditions. Opposing ends of each sample were clamped to the tensiometer using mechanical grips, which moved at a rate of 10 mm/min until the patch broke. The maximum load at which the patch broke was recorded with Merlin IX software. The surface roughness of the patch was assessed using a light interferometry profilometer (GTK1-M Contour, New Spec). Three-dimensional images of the PANI patch were obtained using a 20× objective in vertical scanning interferometry mode. The surface wettability for the PANI patch (*n* = 5) was measured using the sessile drop technique (Ramé-Hart Instrument Co.), and the advancing contact angle was determined using DROPimage Standard software analysis.

#### Electronic characterization.

Cyclic voltammetry measurements were recorded using a CHI660D instrument. The counter electrode was a platinum mesh, and the reference electrode was Ag/AgCl in saturated KCl aqueous solution (CHI Instruments). The working electrode was gold-coated Mylar (VacuLayer Corp.) on which the patches were fabricated. Electrochemical measurements were performed at a scan rate of 25 mV·s^−1^ using 0.1 M PBS aqueous electrolyte solutions. Conductivity of dry films was measured by recording four-probe dc electric measurements using the Keithley 2400 SourceMeasure Unit coupled to a digital multimeter (Agilent 34401A).

To monitor the stability of the PANI patches during incubation in PBS, their surface resistivity was determined at different time points. Rectangular samples (length, 3 cm; width, 0.5 cm; *n* = 3) were fabricated and mounted in custom-made chambers. The chamber (diameter, 1 cm) was filled with PBS. Flat electrodes of gold-coated Mylar were pressed at the edges of the well (1.2 cm apart). The gold electrodes were connected to a source-measuring unit, and the *I-V* characteristics of the patches were recorded by sweeping the voltage at ±1 V. The surface resistivity (ρ) was calculated according to Eq. 1

$$\rho =\frac{w}{L}R$$ (1)

where *R* is the resistance of the patch calculated from the slope of *V* versus *I*, *w* is the width of the film (0.5 cm), and *L* is the distance between the two electrodes (1.2 cm).

To test the electronic stability of the patch in physiological medium, samples fabricated on gold Mylar were assembled in the custom-made chamber filled with PBS and used as the anode. A platinum plate was used as the cathode. A rectangular pulsed regime of 0.1 V, 2 ms at 1 Hz was applied for 2 weeks or a regime of bipolar pulses of ±0.5 V was applied for 1 week using a Stanford Research Systems DS345 function generator. The output voltage and current through the film were measured using a Rigol DS1052E digital oscilloscope, with the current converted to voltage by a Femto DLPCA-200 preamplifier. For cases where the resistance of the patch was low enough to approach the output impedance of the signal generator, custom-made voltage dividers tailored to each particular patch were used to increase the circuit resistance.

#### UV-Vis measurements.

PANI patches (*n* = 4) were fabricated as described above, except for the polymerization time, which was decreased from 3 hours to 5 min to obtain films with reduced opacity, allowing for the transmission of the UV beam. Samples were prepared on glass slides (4 cm × 1 cm) and incubated in a 3-ml cuvette containing PBS. Their absorption spectra were recorded at predetermined time points with a PerkinElmer Lambda 25 UV/Vis spectrophotometer in the range between 300 and 900 nm.

#### X-ray photoelectron spectroscopy.

XPS spectra were obtained on a Thermo Fisher K-Alpha spectrometer using a monochromatic Al-Kα x-ray source (energy, 1486.71 eV), with an electron takeoff angle normal with respect to the analyzer. A low-energy electron/ion flood gun was used to ensure effective surface charge compensation. Survey spectra in the range of 0 to 1300 eV were recorded for each sample (pass energy, 200 eV) followed by high-resolution measurements (pass energy, 50 eV) for C 1*s*, N 1*s*, S 2*p*, P 2*p*, and O 1*s* core levels. Despite the use of the electron flood gun, some minor charging was seen; therefore, spectra were calibrated to the adventitious C 1*s* signal (284.6 eV).

Curve fitting of high-resolution spectra was carried out using Thermo Scientific Avantage (version 5.948) with Shirley background corrections. The amine peak was fixed at 399.2 eV, whereas the positions for the other peaks were variable; however, reasonable peak fits had imine, protonated amine, and protonated imine located at ≈398.3, ≈400.8, and ≈402.1 eV, respectively. Curve fits used pseudo-Voigt functions, which are the product of Lorentzian and Gaussian line shapes, with the percentage of the Lorentzian component variable between 0 and 15%. The full half-width maximum was constrained to be between 1.2 and 2 eV. Peak areas were normalized within the Avantage software using atomic sensitivity factors (*62*, *63*), and from these areas, nitrogen composition, the level of doping, and elemental ratios were determined.

#### Preparation of viable myocardial slices.

Rat left ventricle myocardial slices were prepared using a high-precision vibrating microtome (7000smz, Campden Instruments Ltd.), as described by Camelliti *et al*. (*51*). The 300-μm-thick slices were kept in cold (4°C) and oxygenated (100% O_2_) Tyrode’s solution (140 mM NaCl, 6 mM KCl, 10 mM glucose, 10 mM Hepes, 1 mM MgCl_2_, and 1.8 mM CaCl_2_; pH 7.4) containing the excitation-contraction uncoupler 2,3-butanedione monoxime (BDM; 10 mM). Electrophysiological and contractility recordings were performed in 37°C BDM-free KCl (4.5 mM) Tyrode’s solution.

#### Contractility.

To measure the contractility of stretched myocardial slices, polytetrafluoroethylene-coated silver wire rings were attached to the edges of 300-μm-thick slices perpendicular to the muscle fiber orientation. The samples were then stretched, and the contractility was measured using the HSE isometric force transducer F10 (Hugo-Sachs Electronik/Harvard Apparatus). Force measurements were taken on myocardial slices (*n* = 13); the patches were then positioned on the surface of the slice and removed after the recordings were taken. Different sizes of C-Patches [9 mm^2^ (*n* = 3), 17 mm^2^ (*n* = 4), and 25 mm^2^ (*n* = 13)] and one single size of nC-Patch [25 mm^2^ (*n* = 8)] were assessed on the same cardiac slice. Data are shown as values normalized to the contractility of the slice before the patches were applied.

#### Multielectrode array.

Noninvasive synchronous multifocal recording of extracellular field potentials of myocardial slices was studied using a MEA system (MEA1060, Multichannel Systems). The heart slices were positioned in the center of the MEA dish, and the patches were positioned on the surface of the slice and held in contact by a slice holder. The samples were continuously superfused with oxygenated Tyrode’s solution at 37°C, and bipolar pulses of 1 to 5 V (frequency of 1 Hz) were applied to electrically stimulate the slices (*n* = 7) from different MEA microelectrodes located around the edge of the preparation. This was followed by stimulating the slices with C-Patches of different sizes [9 mm^2^ (*n* = 8) and 25 mm^2^ (*n* = 6)] positioned on top. C-Patches were then removed, and the slices (*n* = 7) were subjected to stimulation again. Field potential recordings were processed to generate activation maps and to measure CV using MC_Rack Software and MATLAB. We refer to CVL and CVT as the maximum and minimum CV correlated with the muscle fiber direction.

#### MI surgical procedure.

All animal procedures were carried out in accordance with the U.K. Home Office Animals (Scientific Procedures) Act 1986 and Directive 2010/63/EU of the European Parliament on the protection of animals used for scientific purposes and approved by the Imperial College Governance Board for Animal Research. Adult male Sprague-Dawley rats (250 to 350 g) were used in this study (*n* = 20). Rats were subjected to acute MI induced by ligation of the LAD. Induction of anesthesia was performed in an induction chamber with 5% isoflurane/95% oxygen. Rats were then intubated, and anesthesia was maintained during surgery with 2% isoflurane/98% oxygen. Rats were given perioperative analgesia [buprenorphine (0.05 to 0.1 mg/kg) and Rimadyl (5 mg/kg)], prophylaxis [Baytril (5 mg/kg)], and 1 ml of 0.9% saline administered subcutaneously. A left parasternal incision followed by a thoracotomy through the fourth intercostal space and removal of the pericardium allowed visualization of the heart. Ligation of the LAD was performed 1 to 2 mm distal to the inferior border of the left atrium using an 8-0 Prolene suture. Effective ligation was confirmed by blanching and cyanosis of the left ventricular free wall and apex. The thoracotomy and chest wall were closed using 4-0 coated Vicryl sutures, and the skin was closed using 4-0 Ethibond Excel sutures. Rats were given daily doses of Rimadyl (5 mg/kg) for at least 48 hours after surgery. Two weeks after LAD ligation, the hearts were explanted and used for whole-heart optical mapping experiments.

#### Whole-heart optical mapping.

To assess the effect of applying a conductive material to the surface of the heart, we used an ex vivo whole-heart epicardial voltage mapping technique to investigate electrical propagation across the epicardium. Optical mapping was carried out on healthy hearts and those that had undergone myocardial infarction 2 weeks previously. The heart was rapidly explanted and rinsed free of blood in ice-cold oxygenated Krebs-Henseleit solution (119 mM NaCl, 4.7 mM KCl, 0.94 mM MgSO_4_, 1 mM CaCl_2_, 1.2 mM KH_2_PO_4_, 25 mM NaHCO_3_, and 11.5 mM glucose and equilibrated with 95% O_2_ + 5% CO_2_) containing heparin (12 U/ml). The heart was transferred to a Langendorff apparatus, where the aorta was cannulated and the heart was retrogradely perfused with oxygenated Krebs-Henseleit solution.

The heart was perfused at a fixed flow rate (15 ml/min) inside a perspex optical mapping chamber. Following a 15-min stabilization period, the heart was stained with the potentiometric dye 4-(2-(6-(dibutylamino)-2-naphthalenyl)ethenyl)-1-(3-sulfopropyl)pyridinium hydroxide [di-4-ANEPPS; 30 μl of di-4-ANEPPS (1 mg/ml) in dimethyl sulfoxide] and perfused with the excitation-contraction uncoupler blebbistatin (10 μM) to eliminate motion artifact. The heart was excited using 530-nm light-emitting diodes, and emitted light was collected using a complementary metal oxide semiconductor camera. Signals were recorded during pacing from the base of the left ventricular free wall (400 beats per min; 1 mA). Action potentials were recorded with no patch on the heart and after the C- or nC-Patch was sutured on. The patches were sutured on with 8-0 Prolene sutures using the minimum number of sutures required to ensure good contact between the material and the epicardial surface.

#### Optical mapping data analysis.

The voltage signal characteristics, including activation time and CV, were extracted using a bespoke MATLAB script. The raw signals were mean-subtracted and inverted, and peaks were detected using a threshold partially set by the user. APD50, APD70, APD90, etc., were calculated as the time between the start of activation to the point on the relaxation slope that corresponded to 50%, 70%, 90%, etc., of the relaxation from the peak value down to the value at the start of the activation (back down to baseline). The conduction velocity was calculated based on methods published by Laughner *et al.* (*52*).

#### Sutureless application of the patch in vivo.

All experiments were performed in accordance with the U.K. Home Office regulations, as stated above. Animals were observed in the operating theater for an hour, following recovery from general anesthesia, at which point any signs of pain or distress were acted upon in accordance with Home Office regulations. Animals were checked twice a day for the first three postoperative days during which analgesia and antibiotics were given. They were weighed daily for the first week to pick up subtle signs of failure to thrive and continued to be checked once a day thereafter.

Male Sprague-Dawley rats between 250 and 350 g were used for all experiments (*n* = 20). Induction of anesthesia was achieved, with the animal placed in an induction chamber and using the inhalation anesthetic agent isoflurane at 3 to 5% with oxygen flow at 3 liters/min. The animal was shaved over the anterolateral aspect of the left chest wall and replaced into the chamber. Once adequate anesthesia was confirmed by assessing pedal reflexes, muscle tone, and respiratory rate, the animal was then intubated using a 16-gauge cannula and mechanically ventilated via a rodent ventilator. It was placed supine on a draped heat mat, with its outstretched limbs taped and its body temperature maintained at about 37°C. Maintenance of anesthesia was achieved with isoflurane at 1.5 to 2% and oxygen flows of 0.4 to 0.5 liter/min. Heart rate and oxygen saturation were monitored throughout the procedure using a rodent pulse oximeter connected to its foot. The animal was prepped with povidone-iodine, and a sterile field was achieved using transparent nonadhesive drapes. Intraoperative pain relief consists of a single subcutaneous dose of Vetergesic (buprenorphine) at 0.12 mg/kg. Adequate hydration was achieved by giving 1-ml bolus of warm saline subcutaneously before an incision was made. A left anterolateral incision was made over the fourth rib, and a thoracotomy was achieved by cutting through the intercostal muscles. The ribs were spread with a self-retaining retractor to expose the beating heart. The parietal pericardium was excised, and the left ventricle was partially extruded from the thoracic cavity. The patch was placed over the left ventricular free wall and secured in place using laser technology (movie S1) (*54*, *55*). Briefly, the area of the patch without PANI was irradiated by a diode-pumped solid-state laser (CNI Lasers). The laser system has a multimode optical fiber (core diameter, 200 μm) inserted into a handheld probe to provide easy and accurate beam delivery. The laser was irradiated at a power of 170 ± 8 mW at 532 nm in a continuous wave with a beam spot diameter on tissue of ~0.6 cm. The C-Patch was spot-irradiated for a total time of 180 ± 9 s to deliver an average fluence of 110 J/cm^2^ (*19*). The nC-Patch was irradiated using the same power and fluence. Laser eye protection goggles were worn during this part of the procedure. The heart was allowed to drop back into the chest cavity, and the lungs were inflated. The ribs and muscle layers were reapproximated using 5-0 Vicryl Rapide sutures, and the skin was closed using a subcuticular approach with 4-0 Monocryl. Isoflurane was turned off, and the animal was allowed to wake while gradually lowering the minute ventilation to stimulate intrinsic ventilatory effort. Postoperative analgesia was achieved by subcutaneously injecting carprofen (Rimadyl) at 5 mg/kg at the end of the operation and once daily for 2 days. Antibiotic cover consisted of enrofloxacin given subcutaneously at 1 mg/kg at the end of the operation and daily for 2 days. The animal was then placed in the recovery chamber until full recovery. The procedure took about 1.5 hours.

#### Echocardiography.

Anesthetized rats were maintained on 1.5% isoflurane. Transthoracic echocardiography was performed by a blinded examiner using a Vevo 2100 (VisualSonics) system. Two-dimensional and M-mode cine loops of a long-axis view and a short-axis view at midlevel of the papillary muscles were recorded. Left ventricular dimensions, FS, and EF were calculated from the M-mode.

#### ECG and arrhythmia provocation.

Anesthetized rats were maintained on 1.5% isoflurane. ECG was recorded using LabChart Pro software and a PowerLab data acquisition system. It is recorded from needle electrodes inserted subcutaneously into the right forelimb and left hind limb. Body temperature was monitored using a rectal probe and maintained at 37°C using an adjustable heat pad. Baseline ECG recording was taken for 15 min. The rat was given an intraperitoneal injection of adrenaline (0.5 mg/kg), and ECG was recorded for 30 min. If no terminal arrhythmia was induced, a second intraperitoneal injection of adrenaline (0.5 mg/kg) was given, and ECG was recorded for a further 30 min. Arrhythmia events were counted according to type (atrial ectopic, ventricular ectopic, couplets, triplets, nonsustained VT, and VF), and each rat was assigned an arrhythmia inducibility score (0 = no arrhythmic events; 1 = minor events such as PVCs, couplets, and triplets; 2 = major events such as nonsustained VT, sustained VT, and VF). Heart rate variability was analyzed using the LabChart Pro HRV add-on with the exclusion of ectopic beats. Baseline heart rate is the average heart rate over the 10 min preceding the first adrenaline injection. The heart rate after adrenaline is taken as the average heart rate over the final 10 min of the procedure.

#### Histology and immunohistochemistry.

For histological staining, cross sections (20 μm) of isopentane-frozen hearts were air-dried and fixed for 5 min in ice-cold acetone before staining with hematoxylin and eosin. Microscopy was performed with a Zeiss Axio Observer inverted wide-field microscope. Immunostaining was performed on 20-μm frozen sections, and the samples were fixed for 5 min in ice-cold methanol and permeabilized for 30 min in PBS with 0.5% Triton X-100. The sections were incubated in blocking solution [1% (w/v) BSA] at room temperature for 1 hour and then were sequentially incubated with primary antibody at 4°C overnight and secondary antibody for 1 hour at room temperature in the dark. Between each staining, the samples were washed three times in PBS (10 min each). Vectashield mounting medium with 4′,6-diamidino-2-phenylindole was used to mount the samples on glass slides that were then stored in slide boxes wrapped in aluminum foil at 4°C. The images were collected using a Zeiss LSM-780 inverted confocal microscope. The antibodies used for immunohistochemistry are summarized in table S2. To quantify the foreign body response to the patches, the size of the cellular capsule was measured. Sections of heart and attached patch at the midmyocardium were stained with PicroSirius red to visualize collagen and Fast Green (Abcam) to label cells and then imaged for analysis. This stain distinguished the healthy myocardium from the fibrotic cellular capsule. The thickness of the capsule was measured at several points, and the width of the ventricle wall was included for comparison.

#### Statistics.

Data are expressed as means ± SE. Unpaired and paired *t* tests were used as appropriate. One-way ANOVA with Tukey post hoc test was used for comparisons of multiple groups. Data that were not normally distributed were compared using a Kruskal-Wallis test with Dunn’s post hoc test. Values of *P* < 0.05 were considered significant.

## Supplementary Material

http://advances.sciencemag.org/cgi/content/full/2/11/e1601007/DC1

## SUPPLEMENTARY MATERIALS

Supplementary material for this article is available at http://advances.sciencemag.org/cgi/content/full/2/11/e1601007/DC1 table S1. Solutions of aniline, phytic acid, and APS containing different molar ratios of aniline to either the dopant or the oxidant. table S2. Primary and secondary antibodies, dilutions, and suppliers. fig. S1. Different forms of PANI. fig. S2. Surface topography imaged by optical profilometer. fig. S3. Cyclic voltammetry at days 1 and 14 of incubation. fig. S4. I-V curves of chitosan and PANI patch. fig. S5. Changes in absorbance at 420 nm and the shift in the polaron region. fig. S6. XPS surface analysis of PANI patch at fabrication. fig. S7. XPS surface analysis of PANI-PCL film at fabrication. fig. S8. Apical and basal APD after cardiac patch attachment ex vivo. fig. S9. Schematic presentation of the sutureless patch application. fig. S10. Representative histological images of patches after 2 weeks in vivo. fig. S11. Representative histological images at different magnifications of patches after 2 weeks in vivo. movie S1. The procedure of the in vivo photoadhesion of the PANI patch using a green laser. movie S2. Strength of the sutureless adhesion after ex vivo photoadhesion to heart tissue.
