# Supplementary material for: A conducting polymer with enhanced electronic stability applied in cardiac models
Source: Sci Adv. 2016 Nov 30;2(11):e1601007. doi: 10.1126/sciadv.1601007 (PMC5262463; doi:10.1126/sciadv.1601007)
Supplement: http://advances.sciencemag.org/cgi/content/full/2/11/e1601007/DC1 [file supp_2_11_e1601007__index.html]

Science Advances | Science Advances

## Supplementary Materials

**This PDF file includes:**

- table S1. Solutions of aniline, phytic acid, and APS containing different molar ratios of aniline to either the dopant or the oxidant.
- table S2. Primary and secondary antibodies, dilutions, and suppliers.
- fig. S1. Different forms of PANI.
- fig. S2. Surface topography imaged by optical profilometer.
- fig. S3. Cyclic voltammetry at days 1 and 14 of incubation.
- fig. S4. *I-V* curves of chitosan and PANI patch.
- fig. S5. Changes in absorbance at 420 nm and the shift in the polaron region.
- fig. S6. XPS surface analysis of PANI patch at fabrication.
- fig. S7. XPS surface analysis of PANI-PCL film at fabrication.
- fig. S8. Apical and basal APD after cardiac patch attachment ex vivo.
- fig. S9. Schematic presentation of the sutureless patch application.
- fig. S10. Representative histological images of patches after 2 weeks in vivo.
- fig. S11. Representative histological images at different magnifications of patches after 2 weeks in vivo.
- Legends for movies S1 and S2

Download PDF

**Other Supplementary Material for this manuscript includes the following:**

- movie S1 (.wmv format). The procedure of the in vivo photoadhesion of the PANI patch using a green laser.
- movie S2 (.mp4 format). Strength of the sutureless adhesion after ex vivo photoadhesion to heart tissue.

**Files in this Data Supplement:**

- Adobe PDF - 1601007\_SM.pdf
