# Supplementary material for: A conducting polymer with enhanced electronic stability applied in cardiac models
Source: Sci Adv. 2016 Nov 30;2(11):e1601007. doi: 10.1126/sciadv.1601007 (PMC5262463; doi:10.1126/sciadv.1601007)
Supplement: http://advances.sciencemag.org/cgi/content/full/2/11/e1601007/DC1 [file 1601007_SM.pdf]

Science Advances
AAAS
advances.sciencemag.org/cgi/content/full/2/11/e1601007/DC1

Supplementary Materials for

A conducting polymer with enhanced electronic stability applied in cardiac models

Damia Mawad, Catherine Mansfield, Antonio Lauto, Filippo Perbellini, Geoffrey W. Nelson, Joanne Tonkin, Sean O. Bello, Damon J. Carrad, Adam P. Micolich, Mohd M. Mahat, Jennifer Furman, David Payne, Alexander R. Lyon, J. Justin Gooding, Sian E. Harding, Cesare M. Terracciano, Molly M. Stevens

Published 30 November 2016, *Sci. Adv.* **2**, e1601007 (2016)
DOI: 10.1126/sciadv.1601007

The PDF file includes:

- table S1. Solutions of aniline, phytic acid, and APS containing different molar ratios of aniline to either the dopant or the oxidant.
- table S2. Primary and secondary antibodies, dilutions, and suppliers.
- fig. S1. Different forms of PANI.
- fig. S2. Surface topography imaged by optical profilometer.
- fig. S3. Cyclic voltammetry at days 1 and 14 of incubation.
- fig. S4. *I-V* curves of chitosan and PANI patch.
- fig. S5. Changes in absorbance at 420 nm and the shift in the polaron region.
- fig. S6. XPS surface analysis of PANI patch at fabrication.
- fig. S7. XPS surface analysis of PANI-PCL film at fabrication.
- fig. S8. Apical and basal APD after cardiac patch attachment ex vivo.
- fig. S9. Schematic presentation of the sutureless patch application.
- fig. S10. Representative histological images of patches after 2 weeks in vivo.
- fig. S11. Representative histological images at different magnifications of patches after 2 weeks in vivo.
- Legends for movies S1 and S2

Other Supplementary Material for this manuscript includes the following:
(available at advances.sciencemag.org/cgi/content/full/2/11/e1601007/DC1)

- movie S1 (.wmv format). The procedure of the in vivo photoadhesion of the PANI patch using a green laser.

- movie S2 (.mp4 format). Strength of the sutureless adhesion after ex vivo photoadhesion to heart tissue.

table S1. Solutions of aniline, phytic acid, and APS containing different molar ratios of aniline to either the dopant or the oxidant.

Group (n=3)	Molar ratio (aniline/phytic acid)	Molar ratio (aniline/APS)	Variable	Surface Resistivity (kilohms per square)*
**Group 1**	7.24	4.00	Recipe reported in the manuscript	5.31 ± 1.64
**Group 2**	3.62	4.00	Increasing the dopant	5.83 ± 2.83
**Group 3**	1.00	4.00	Increasing the dopant	6.57**
**Group 4**	7.24	2.00	Increasing the oxidant	2.15 ± 0.56

*No significant difference (One-way ANOVA analysis, p=0.116) was obtained between the surface resistivity of the 3 groups, suggesting that increasing either the dopant or the oxidant ratio had no effect on the conductivity of PANI.

**films were very brittle. They cracked while attaching them to the electrodes for IV recordings. No reliable measurements could be obtained.

table S2. Primary and secondary antibodies, dilutions, and suppliers.

Primary antibody	Manufacturer	Dilution
Vimentin	Thermoscientific (PA1-10003)	1:5000
Smooth muscle actin (SMA)	Dako (M0851)	1:1500
Von Willebrand Factor	Abcam (ab6994)	1:1000
Alpha sarcomeric actinin	Sigma (A7811)	1:2000

Secondary antibody	Manufacturer	Dilution
Alexa Fluo 568 - Goat anti chicken	Life Technologies (A11041)	1:2000
Alexa Fluo 633 - Goat anti mouse	Life Technologies (A21057)	1:2000
Alexa Fluo 488 - Donkey anti rabbit	Life Technologies A21206)	1:2000
Alexa Fluo 488 - Donkey anti mouse	Life Technologies (A21202)	1:2000

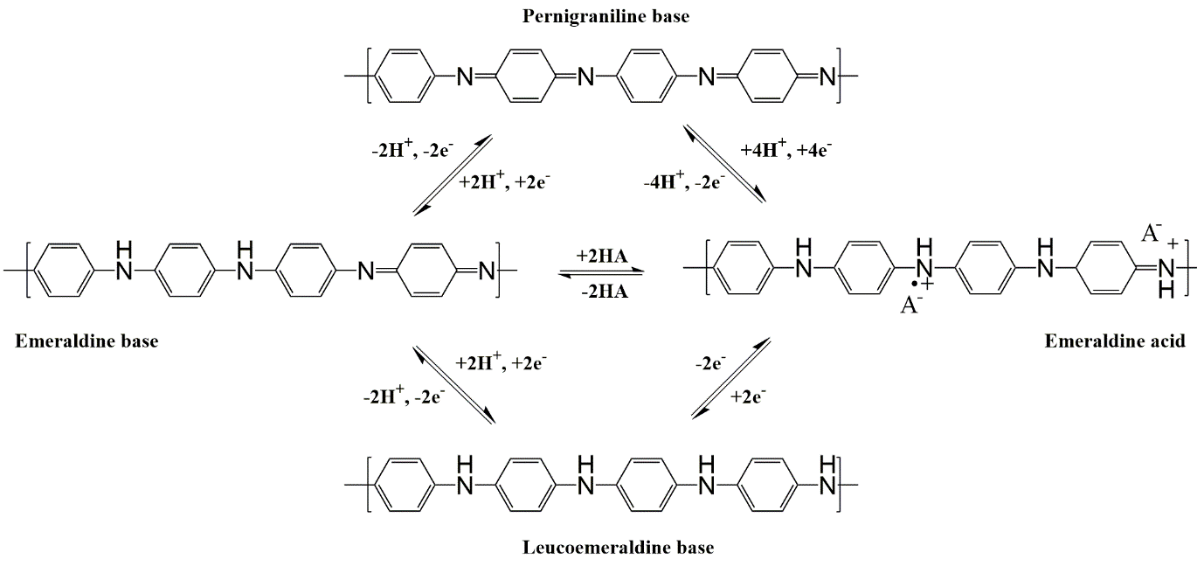

fig. S1. Different forms of PANI. Different forms of polyaniline: fully oxidised form (**pernigralinine base**), fully reduced form (**leucoemeraldine base**), intermediate state with equal number of oxidised and reduced repeat units (**emeraldine base**), and the conductive form induced by protonation (**emeraldine salt**). **A^-** represents the counter ion of the acid introduced for doping (in our case, the phytate group).

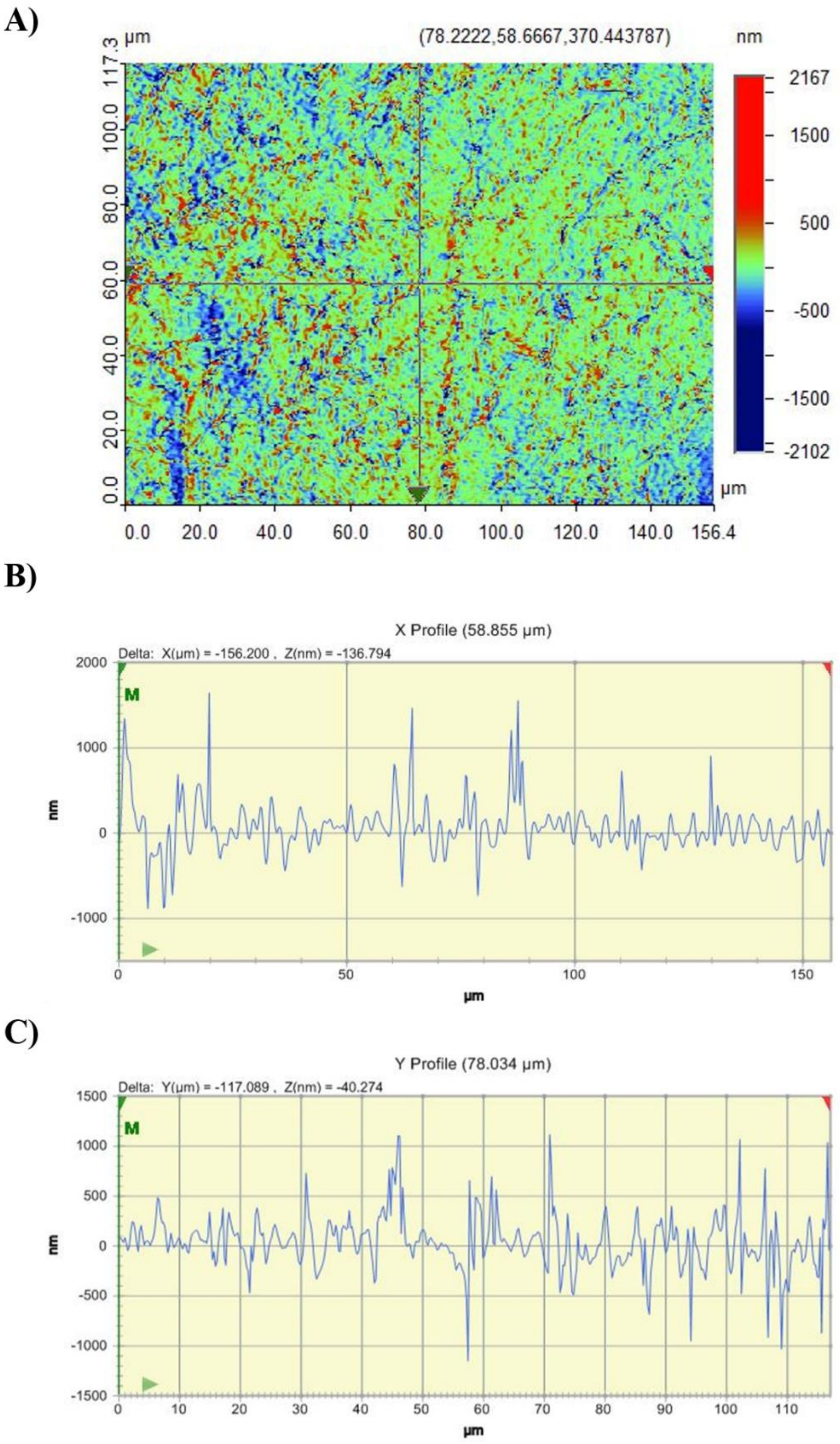

fig. S2. Surface topography imaged by optical profilometer. Optical profilometer was used to image the surface topography of the PANI patch over an area of 117 µm x 156 µm **A**). The surface presents with homogeneous features both in the x **B**) and y **C**) directions.

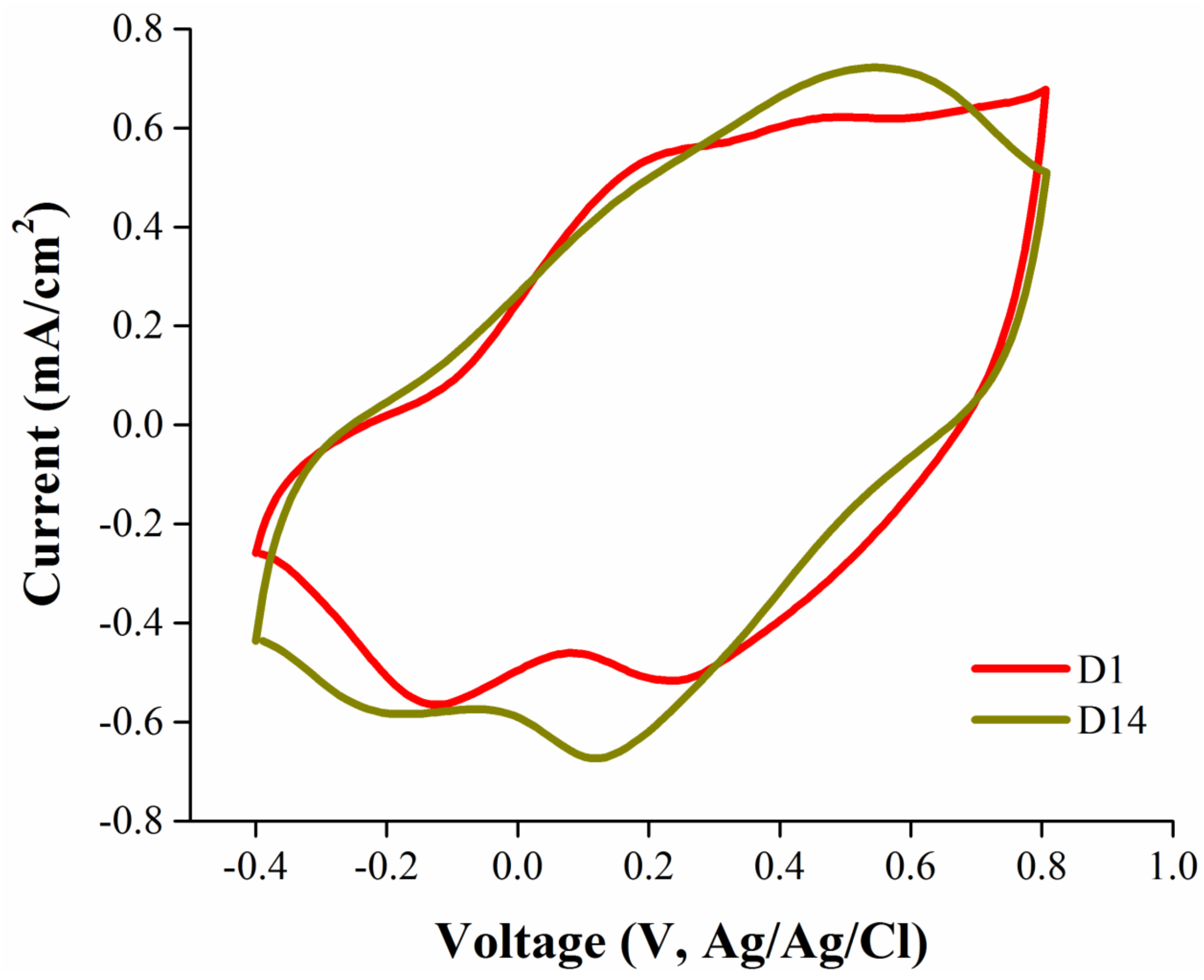

fig. S3. Cyclic voltammetry at days 1 and 14 of incubation. Cyclic voltammograms (25 mV.s^{-1}) after 1 and 14 days incubation in 0.1 M PBS, to highlight the cathodic shift over the incubation period.

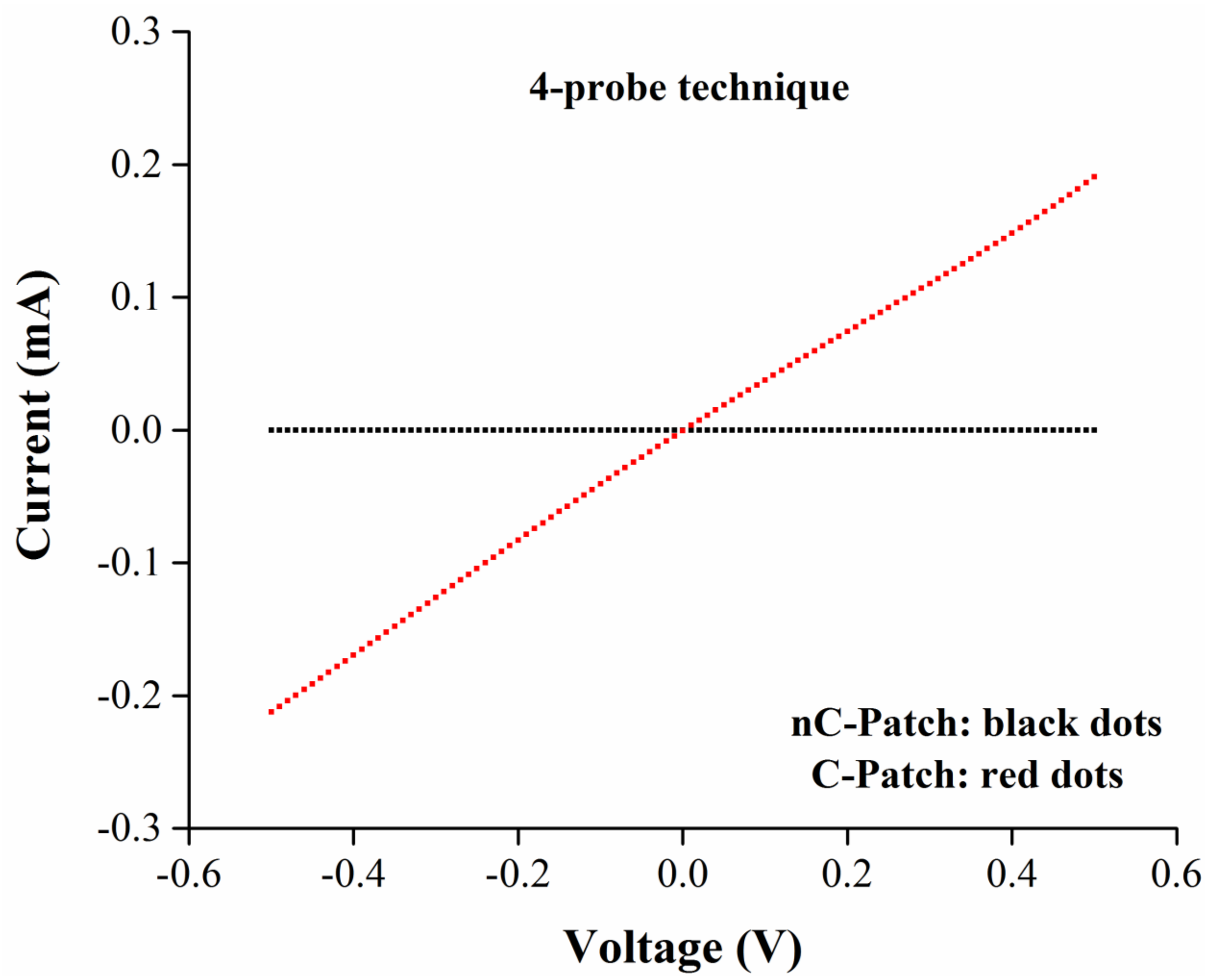

fig. S4. *I-V* curves of chitosan and PANI patch. IV curves measured by the 4-probe technique of dry chitosan film (black) and PANI patch (red).

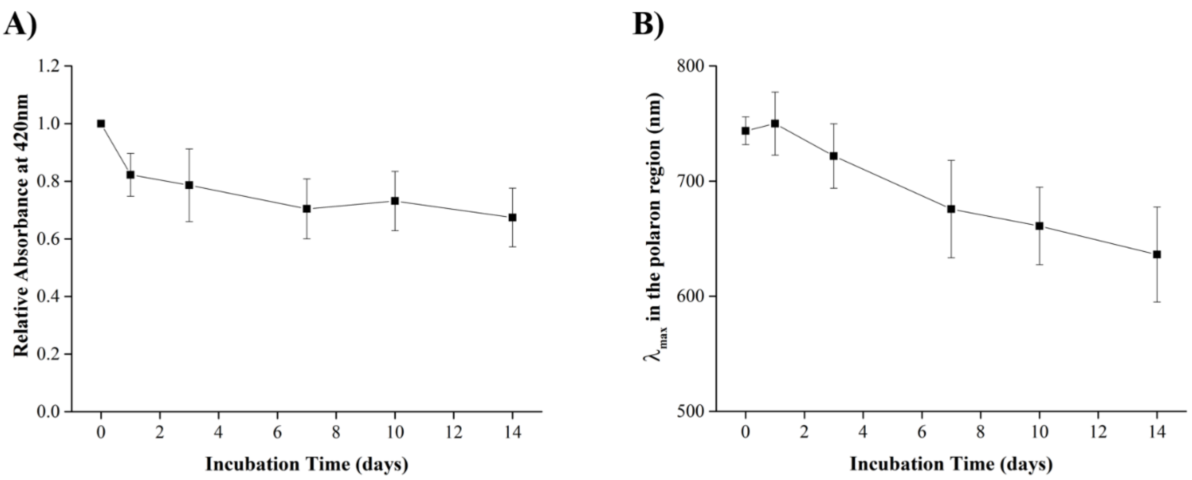

fig. S5. Changes in absorbance at 420 nm and the shift in the polaron region. Changes in **A**) absorbance at λ = 420 nm and **B**) the shift of the maximum peak in the polaron region (500-900 nm) of PANI patch (n = 3) incubated for 2 weeks.

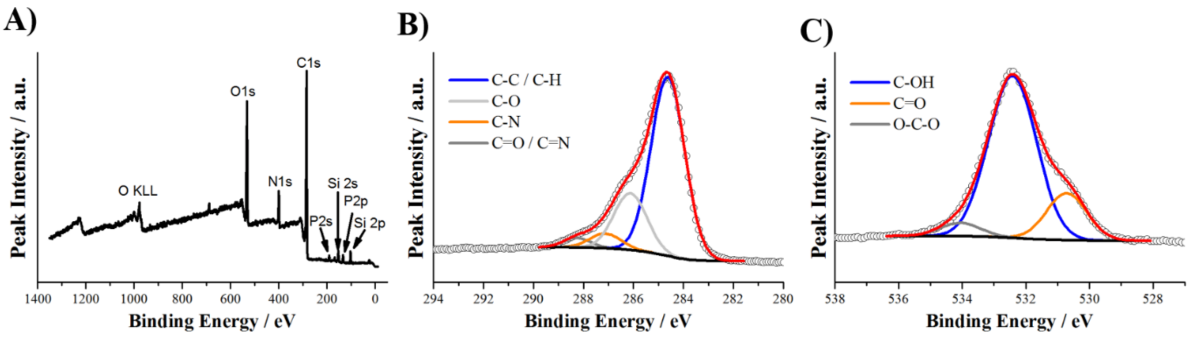

fig. S6. XPS surface analysis of PANI patch at fabrication. XPS results for PANI patch at fabrication with photoelectron peaks identified and curve fitting shown. **A**) Survey; **B**) C 1*s*; **C**) O 1*s*.

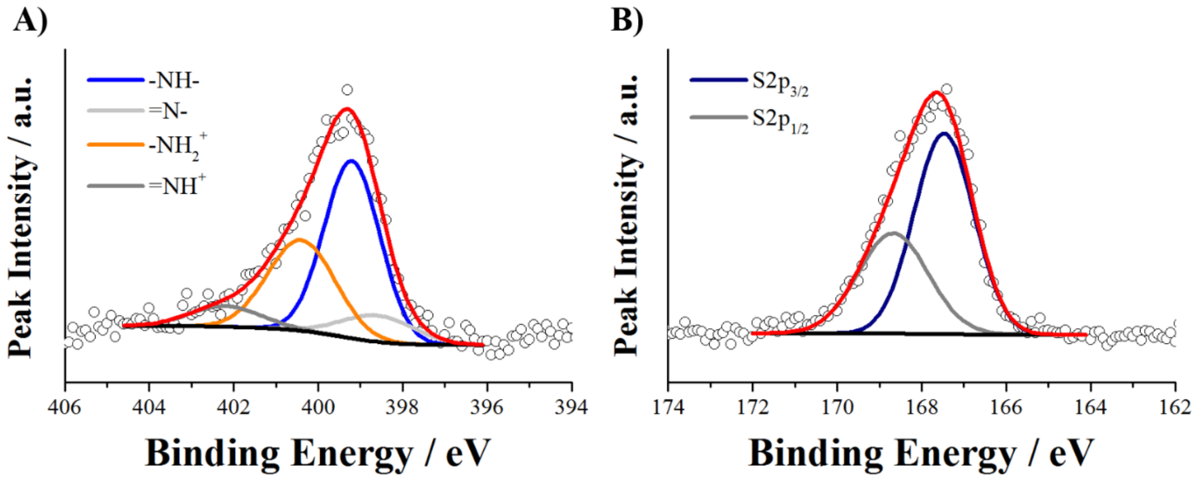

fig. S7. XPS surface analysis of PANI-PCL film at fabrication. XPS results for PANI-PCL at fabrication with photoelectron peaks identified and curve fitting shown. **A**) N 1*s*; **B**) S 2*p*.

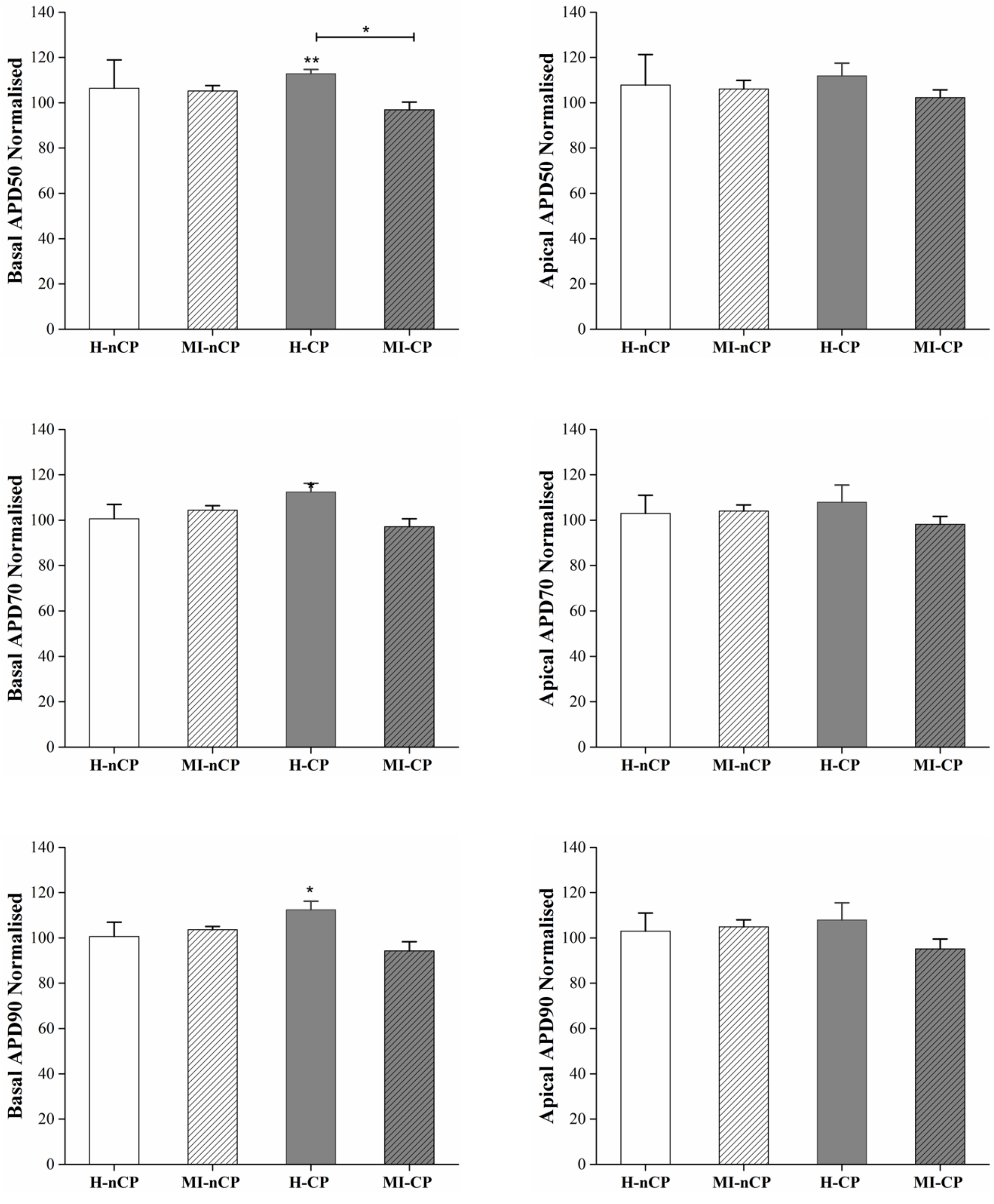

fig. S8. Apical and basal APD after cardiac patch attachment ex vivo. As calculated from whole heart epicardial voltage mapping ($n = 5$; $*p < 0.05$, $**p < 0.005$). Data are normalised to baseline (before patch attachment). APD 50, 70 and 90 = time to 50%, 70% and 90% repolarisation. H: Healthy; MI: Myocardial Infraction; nCP: non-conductive patch; CP: conductive patch.

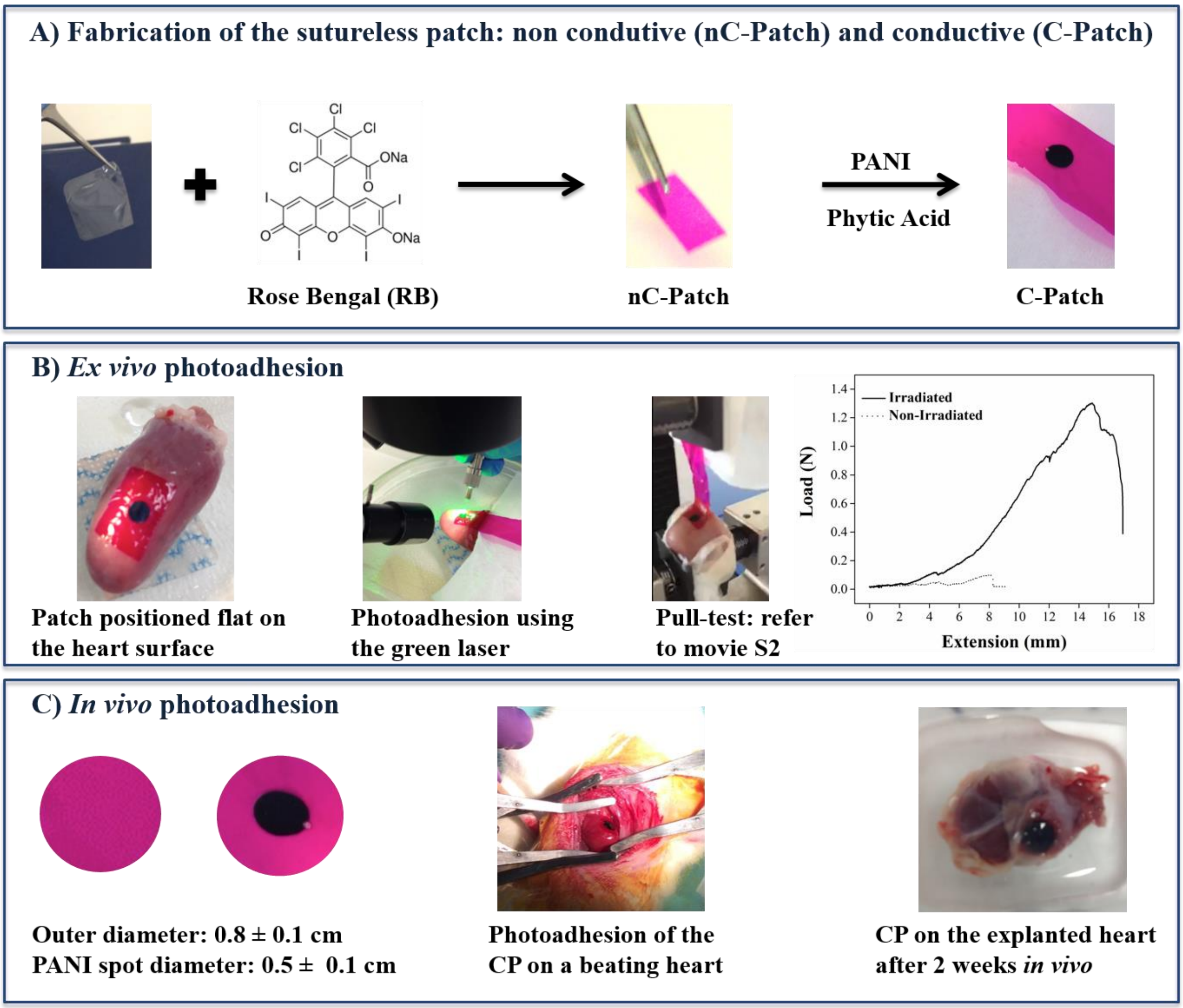

fig. S9. Schematic presentation of the sutureless patch application. A) Rose Bengal is added in the patch to produce a film that can be photoactivated. Control patch: nC-Patch; conductive patch: C-Patch. **B**) The photoadhesion of the patch was tested *ex vivo* on chicken hearts. The patch is positioned flat on the tissue. A green laser is used to spot irradiate the patch (patch area = 0.8 cm^2, irradiation time ~ 9 minutes). The photoadhesion was tested by a pull test (refer to the movie S2). Mechanical testing determined the higher load that the photo-adhered patch can sustain. **C**) Digital photos showing the geometry of the patches prepared for the *in vivo* experiments, the patch photo-adhered on a beating heart and still in position after 2 weeks implantation *in vivo*.

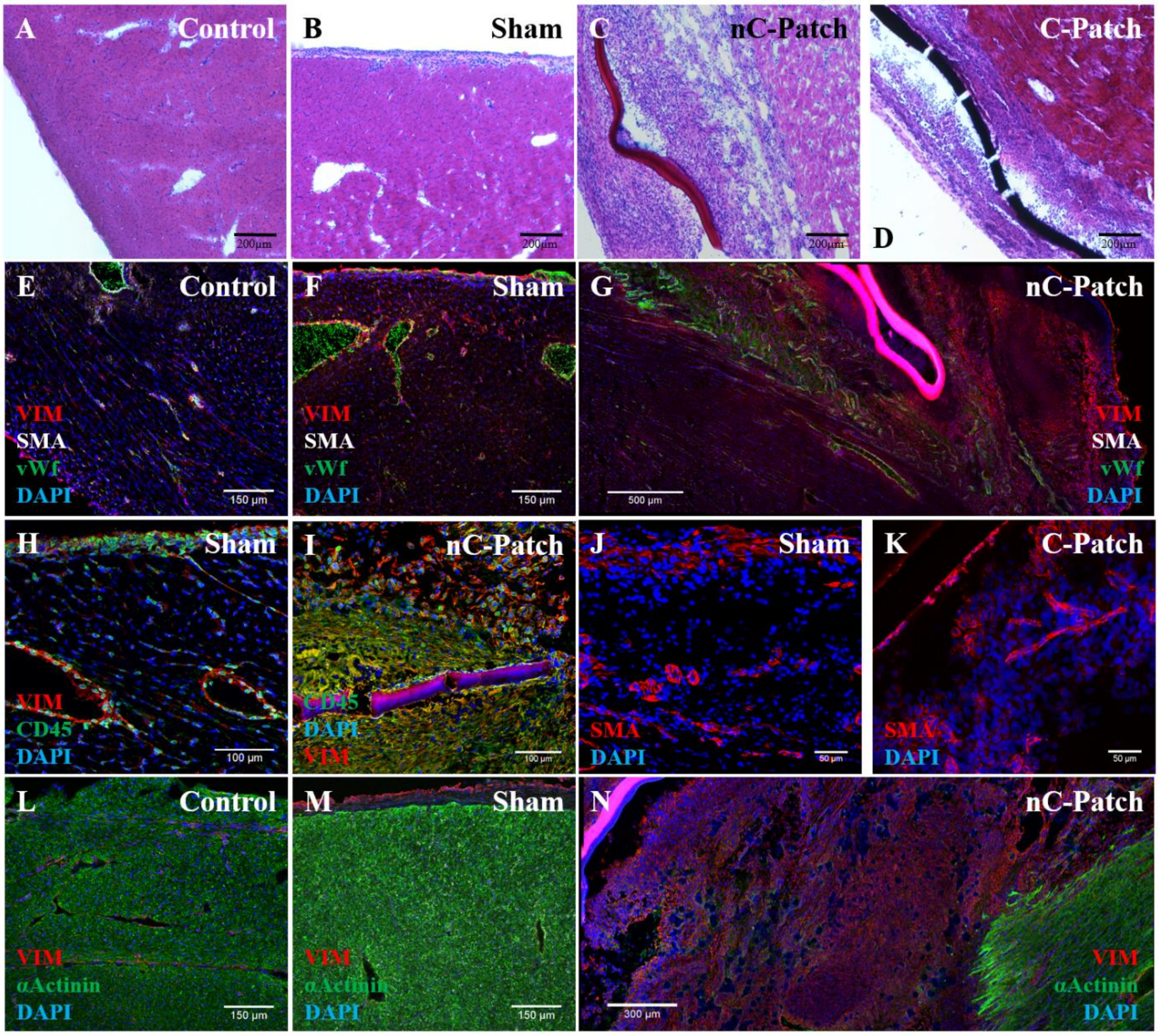

fig. S10. Representative histological images of patches after 2 weeks in vivo. The hearts with both patches, conductive (C-Patch) and non-conductive (nC-Patch), were surrounded by a thicker cellular infiltrate compared to control and sham hearts (**A-D**). Immunostaining for von Willerbrand Factor (vWf) revealed neovascularization in close proximity to the patch (**G**). The cellular infiltrate of sham, nC-Patch and C-Patch was composed of fibroblasts (Vim+) and inflammatory cells (CD45+) (**H**, **I**). In sham samples, the inflammatory cells (CD45+) are mostly present inside the vessels and in the fibrotic layer surrounding the heart (H); in the presence of C-Patch or nC-Patch, these cells surround the biomaterial (I). Many fibroblasts were also SMA+ suggesting the presence of myofibroblats in the fibrotic tissue surrounding the hearts and the patches (**J**, **K**). Despite the presence of fibrotic tissue (Vim+), the cardiomyocytes (α-Actinin positive) appeared undamaged and organized, with a well-defined border myocardium/scar (**L-N**).

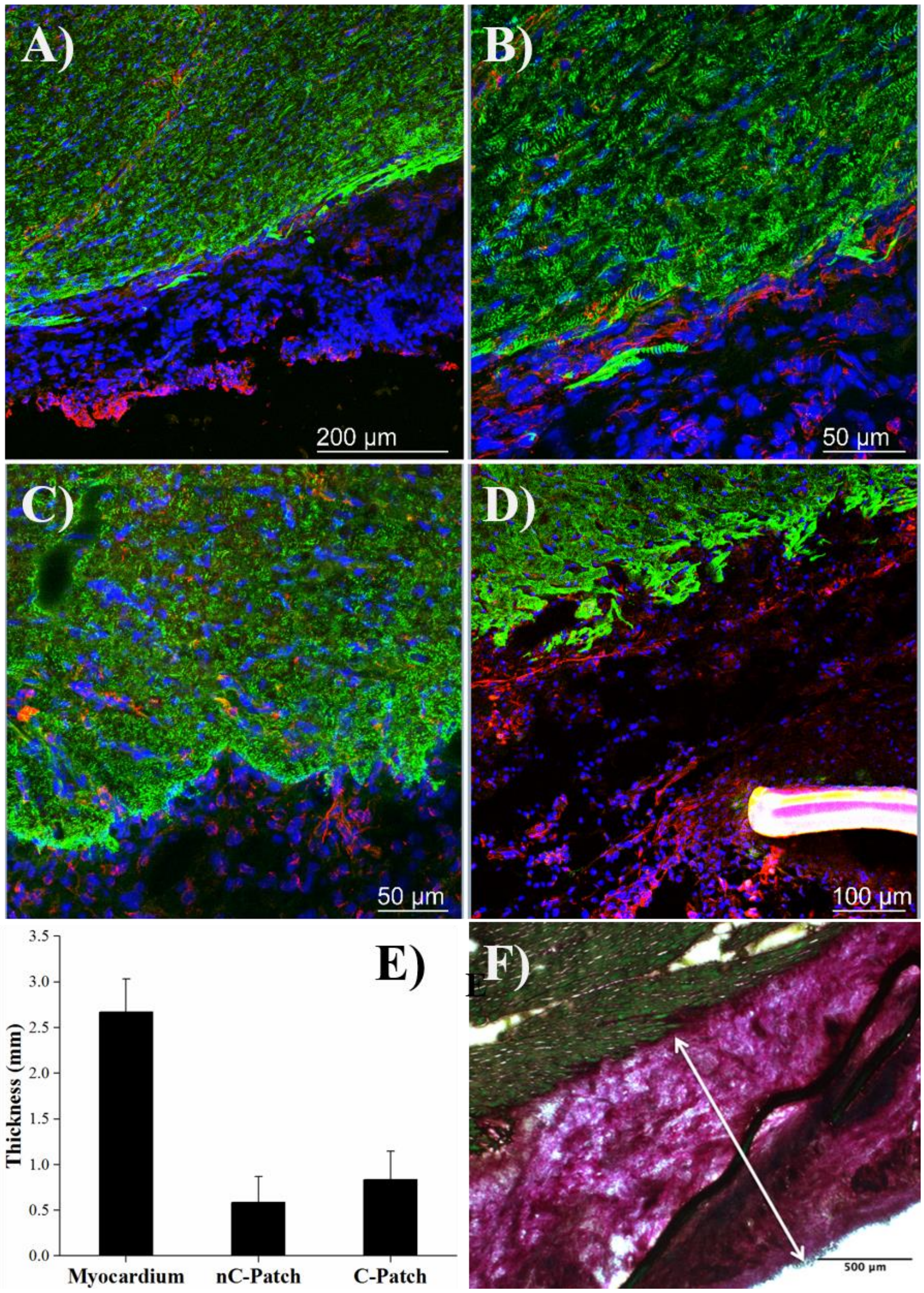

fig. S11. Representative histological images at different magnifications of patches after 2 weeks in vivo. A) and **B**) Despite the presence of fibrotic tissue (Vim+ cells) surrounding the heart, the cardiomyocytes (α-Actinin+ cells) appeared undamaged and organized, with a well-defined border myocardium/scar and no infiltration of fibroblasts within the myocardium. **C**) Acquisition of the tissue near the scar and **D**) the scar that separates the polymer from the

myocardium. **E**) To quantify the foreign body response to the patches, the thickness of the cellular capsule surrounding nC-patch and C-Patch was quantified. The width of the myocardial wall is included for reference. **F**) Staining with PicroSirius red to visualize collagen and Fast Green (Abcam) to label cells was conducted and the sections were used to quantify the thickness.

movie S1. The procedure of the in vivo photoadhesion of the PANI patch using a green laser. This movie is a recording of the sutureless application of the PANI patch on the heart *in vivo*. The patch is positioned on the beating heart and flattened to ensure a good contact with the tissue. The pink area of the patch (around the conductive area) is spot irradiated using the green laser. The heart is then dropped in the cavity.

movie S2. Strength of the sutureless adhesion after ex vivo photoadhesion to heart tissue. This movie is a demonstration of the strong adhesion of the patch to heart tissue following laser irradiation. After spot irradiating a patch on a chicken heart (*ex vivo*) using a green laser to induce photoadhesion, the heart was transfeered to an Instron machine and the extended part of the patch was clamped and pulled upwards. As the patch was extended, it started pulling the tissue with it demonstrating the strength of the photoadhesion technique.
